# Two Antagonistic MALT1 Auto-Cleavage Mechanisms Reveal a Role for TRAF6 to Unleash MALT1 Activation

**DOI:** 10.1371/journal.pone.0169026

**Published:** 2017-01-04

**Authors:** Stefanie Ginster, Maureen Bardet, Adeline Unterreiner, Claire Malinverni, Florian Renner, Stephen Lam, Felix Freuler, Bertran Gerrits, Johannes Voshol, Thomas Calzascia, Catherine H. Régnier, Martin Renatus, Rainer Nikolay, Laura Israël, Frédéric Bornancin

**Affiliations:** Novartis Institutes for BioMedical Research, Novartis Campus, Basel, Switzerland; Johns Hopkins School of Medicine, UNITED STATES

## Abstract

The paracaspase MALT1 has arginine-directed proteolytic activity triggered by engagement of immune receptors. Recruitment of MALT1 into activation complexes is required for MALT1 proteolytic function. Here, co-expression of MALT1 in HEK293 cells, either with activated CARD11 and BCL10 or with TRAF6, was used to explore the mechanism of MALT1 activation at the molecular level. This work identified a prominent self-cleavage site of MALT1 isoform A (MALT1A) at R781 (R770 in MALT1B) and revealed that TRAF6 can activate MALT1 independently of the CBM. Intramolecular cleavage at R781/R770 removes a C-terminal TRAF6-binding site in both MALT1 isoforms, leaving MALT1B devoid of the two key interaction sites with TRAF6. A previously identified auto-proteolysis site of MALT1 at R149 leads to deletion of the death-domain, thereby abolishing interaction with BCL10. By using MALT1 isoforms and cleaved fragments thereof, as well as TRAF6 WT and mutant forms, this work shows that TRAF6 induces N-terminal auto-proteolytic cleavage of MALT1 at R149 and accelerates MALT1 protein turnover. The MALT1 fragment generated by N-terminal self-cleavage at R149 was labile and displayed enhanced signaling properties that required an intact K644 residue, previously shown to be a site for mono-ubiquitination of MALT1. Conversely, C-terminal self-cleavage at R781/R770 hampered the ability for self-cleavage at R149 and stabilized MALT1 by hindering interaction with TRAF6. C-terminal self-cleavage had limited impact on MALT1A but severely reduced MALT1B proteolytic and signaling functions. It also abrogated NF-κB activation by N-terminally cleaved MALT1A. Altogether, this study provides further insights into mechanisms that regulate the scaffolding and activation cycle of MALT1. It also emphasizes the reduced functional capacity of MALT1B as compared to MALT1A.

## Introduction

**Fig 1:**
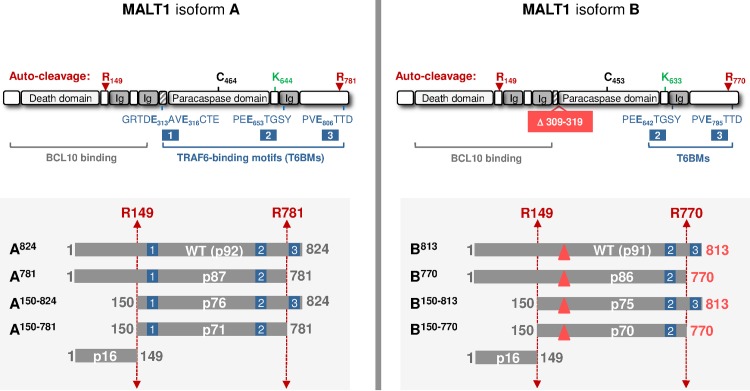
Schematic representation of MALT1 isoforms and variants thereof. The two reported human MALT1 isoforms A (NP_006776.1) and B (NP_776216.1) are depicted. The drawings show the different domains, the auto-cleavage sites (red arrows), the catalytic cysteine residue (black), the reported ubiquitination site (green) and shows the regions required for binding BCL10 (grey) and TRAF6 (blue). The truncated variants of the two isoforms, used or discussed in the present work, are represented in the shaded boxes at the bottom, with predicted molecular weights. The 11-amino acid deletion of isoform B is depicted by a red triangle. Ig, Immunoglobulin-like domain.

Mucosa associated lymphoid tissue lymphoma translocation protein 1(MALT1) also known as paracaspase 1 (PCASP1) [1] plays an important role during immune responses triggered by antigen receptors and C-type lectin receptors such as Dectins [2, 3]. After engagement of the B cell receptor (BCR), the T cell receptor (TCR) or Dectin-1/2, phosphorylation of the relevant CARD protein (CARD11, also known as CARMA1, in lymphocytes; CARD9 in myeloid cells) triggers a conformational change. This enables the specific CARD protein to act as a nucleation center for building, together with pre-assembled BCL10-MALT1 complexes, a multimeric filament-like activated “CBM” complex in the cell [4]. The CBM complex is an essential scaffolding platform that connects via TRAF6 to the inhibitor of kappa B kinase (IKK) complex, leading to canonical NF-κB activation. The CBM complex also regulates other pathways like JNK and p38 signaling [5]. The critical function of the CBM was exemplified in a number of studies in the mouse where knocking out any of its components led to blockade of NF-κB activation following immune receptor stimulation, resulting in poor immune responses [6]. Human patients with CBM deficiencies were also identified all of whom displayed combined immuno-deficiencies [7, 8]. Beyond this essential scaffolding role, MALT1 was shown to have arginine directed proteolytic activity triggered by CBM complex assembly [9, 10]. Several MALT1 proteolytic substrates have been identified, including ubiquitination regulators like A20 [9], CYLD [11] and HOIL-1 [12–14], the NF-κB subunit RELB [15] or mRNA turnover regulators like Regnase-1 [16], and Roquins [17]. In addition, the CBM component BCL10 was shown to be a MALT1 substrate [10]. While the understanding of MALT1 biology is expanding with the identification of new substrates, the mechanisms of MALT1 protease activation have not been fully elucidated. In addition to cleaving substrates, MALT1 was recently reported to undergo N-terminal auto-cleavage after R149, a mechanism shown to be important for NF-κB transcriptional activation [18]. Mono-ubiquitination at K644 was also reported to be a key post-translational modification required for sustained MALT1 activity [19]. Furthermore, two isoforms of MALT1 have been reported. Isoform B is shorter than A, missing 11 residues encoded by exon 7. Alternative splicing of MALT1 in T lymphocytes was recently shown to be a regulated process and a suppressor of exon 7 inclusion was identified [20]. Remarkably, exon 7 encodes one key TRAF6-binding motif (T6BM) between the second immunoglobulin-like domain and the paracaspase domain which is consequently missing in MALT1B (Fig 1).

In this report, we have investigated further the changes occurring in MALT1 following activation, by comparing the two isoforms MALT1A and MALT1B (Fig 1). We have identified a novel C-terminal auto-cleavage site at R781 (MALT1A)/R770 (MALT1B) and demonstrate its critical impact on MALT1 B. This work confirms that MALT1 undergoes mono-ubiquitination after assembly into activating complexes. Although TRAF6 does not appear to be responsible for mono-ubiquitination of MALT1, interaction between TRAF6 and MALT1 has proved essential for auto-proteolytic cleavage of MALT1 at R149 and overall MALT1 protein turnover.

## Materials and Methods

### Expression plasmids

MALT1A and mutants thereof were cloned into pCMVSport, encoding an N-terminal FLAG-His-Strep tag. BCL10 and CARD11-L244P were cloned into pcDNA6.2/V5-DEST (Invitrogen). The TRAF6-expressing plasmid was described previously [21] as well as the plasmid encoding A20 [22]. The plasmids for expression of CYLD, MALT1 isoform B and TRAF2 were obtained from GeneCopoeia™ (pReceiver-M11 vector) as well as the plasmid encoding TRAF3 (pReceiver-M12 vector). The TRAF6-binding deficient MALT1 construct (MALT1-E4/A, N-ter. FLAG-tagged) was obtained from the BCCM/LMBP plasmid collection. Oligonucleotides for mutagenesis reactions are described elsewhere (S1 Table).

### Mutagenesis procedure

Mutagenesis was done using QuikChange II XL Site-Directed Mutagenesis kit (Agilent Technologies, QuikChange manual). Primers encoding the mutation of interest were designed using the Agilent technologies online tool (QuikChange primer design). Template plasmids were mutagenized by PCR amplification in the presence of mutagenesis primers according to the manufacturer’s recommendations. Digestion of the parental methylated strain was performed with DpnI, the mutated plasmids were transformed into XL10 Gold-Ultracompetent Cells, plated on LB plates containing the selection antibiotics (Invivogen) and the plates incubated for 18 hours at 37°C. Plasmids were isolated using Mini- or Maxiprep kits (Qiagen) and sequenced with an Applied Biosystem 96-capillary array sequencer (ABI3730xl) using ABI Cycle Sequencing Chemistry with Dye Terminators.

### Antibodies for immunoblotting

The following antibodies were used: anti-MALT1 (Santa Cruz H-300 #28246, rabbit polyclonal, epitope corresponding to amino acids 525–824), anti-MALT1 [MT1/410] (Abcam ab178581, mouse monoclonal), anti-MALT1 [ep603y] (Abcam ab33921, rabbit monoclonal, epitope corresponding to amino acids 1–100), anti- BCL10 [ep605y] (Abcam ab40752, rabbit monoclonal, reacts with total BCL10, i.e full length + cleaved by MALT1), anti-BCL10 [ep606y] (Abcam ab33905, rabbit monoclonal, reacts preferentially with full-length BCL10), anti-A20 (Abcam, #ab92324, rabbit polyclonal), anti-CARD11 (Cell signaling Technology #4440, rabbit polyclonal), anti-FLAG (Sigma F3165, mouse monoclonal), anti-ubiquitin (Enzo BML-PW8810, mouse monoclonal), anti-TRAF6 [D21G3] (Cell Signaling Technology, #8028, rabbit monoclonal), anti-tubulin-α [B-5-1-2] (Sigma T6074, mouse monoclonal), secondary goat anti-mouse labelled with Alexa Fluor^®^ 680 (ThermoFisher A21059), secondary goat anti-rabbit labelled with IRDye 800CW (LI-COR 926–32211). An affinity-purified rabbit polyclonal antibody was raised against the C-terminal BCL10 neo-epitope (FLPLRSR) generated upon cleavage by MALT1 (Squarix).

### HEK293 cell experiments

293T/17 [HEK 293T/17] (ATCC^®^ CRL-11268™) cells were maintained in DMEM/ GlutaMAX™ (Gibco) containing 4.5 g/L D-Glucose and 25 mM HEPES and supplemented with 10% heat-inactivated fetal calf serum (PAA, A15-152, lot #A15211-0991) and 100 U penicillin / streptomycin. In a 24-well tissue culture treated plate, 150’000 cells/well were transfected with 0.25 μg total DNA (100 ng/ml, diluted in OptiMEM, Gibco) and 1.5 μl X-tremeGENE™ 9 DNA (Roche). To inhibit MALT1 proteolytic function, 100 μM z-VRPR-fmk (dissolved in 1:1 DMSO/water) was added to the cells 5 h after transfection. Twenty hours later cells were washed with cold PBS containing protease inhibitor (Roche) and phosphatase inhibitor (Sigma) cocktails and then lysed in 100 μl lysis buffer (150 mM NaCl, 1 mM Na_2_EDTA, 1 mM EGTA, 1% Triton, 2.5 mM sodium pyrophosphate, 1 mM β-glycerophosphate, 1 mM Na_3_VO_4_, 1 μg/ml leupeptin, Cell Signaling Technology). Finally, 100 μl lysates were transferred into cold microtubes, mixed with an equal volume of 4x-LDS / Reducing NuPAGE^®^ sample buffer (Thermo Fisher) and denaturated at 95°C.

### OCI-Ly3 cell experiments

OCI-Ly3 cells [23] were cultured in RPMI 1640/GlutaMAX™ (Gibco), supplemented with 20% heat-inactivated fetal calf serum and 100 U penicillin / streptomycin (Gibco). For immunoprecipitation analysis of MALT1 the B lymphoma cells were treated during four days with 50 μM z-VRPR-fmk (dissolved in 1:1 DMSO/water) or vehicle. The MALT1 inhibitor was added on the first day and once again on the second day. Full cell lysates were then obtained using cold buffer containing 50 mM β-glycerophosphate at pH7.5–1% NP40–0.5% Na cholate—0.1% SDS– 2 mM DTT, cOmplete™ protease (Roche) and phosphatase inhibitors cocktails 2 & 3 (Sigma). The lysates were then treated ± with Dynabeads^®^ Protein G (Invitrogen) coupled with anti MALT1 C-ter (H-300, Santa Cruz) according to the manufacturer guidelines. The eluted fractions, as well as the full lysates, were mixed with 1x-LDS / Reducing NuPAGE^®^ Sample buffer and denatured at 95°C.

### Jurkat cell experiments

Jurkat cells were maintained in RPMI1640/ GlutaMAX™ (Gibco) supplemented with 10% heat-inactivated fetal calf serum (PAA, A15-152, lot #A15211-0991) and 100 U penicillin / streptomycin, HEPES (5ml) and Sodium Pyruvate MEM 1mM + D+ Glucose solution (0.45%). One day before the experiment, cells were diluted two-fold. On the next day, 2 ml of cells at 1,200,000 cells/ml were added to a well of a 6-well plate and were treated with z-VRPR-fmk (dissolved in 1:1 DMSO/water) at 50 μM. Thirty minutes later, cells were stimulated with various stimuli e.g., PMA (Sigma, 10 ng/ml), anti-CD3 antibody ([OKT3], Novartis, 1 μM) and anti-CD28 antibody ([15E8], Novartis, 1 μM), or PMA (10 ng/ml) and Ionomycin (Sigma, 1 μM) for increasing times, as described in the figure legends. The proteasome inhibitor MG-132 (Sigma, 5 μM) was also added in some experiments. At the end of the time course, cells were washed with cold PBS containing protease and phosphatase inhibitor cocktails and then lysed in 100 μl lysis buffer (Cell Signaling Technology + protease inhibitors). An equal volume of 2x-LDS / Reducing NuPAGE^®^ sample buffer (Thermo Fisher) was added before denaturation at 95°C.

### Primary human T cell experiments

Human PBMCs were isolated from buffy coats by Ficoll-Paque™ density separation. Red blood cells were lysed for 5 min at RT with 150 mM NH_4_Cl, 10 mM KHCO_3_ and 0.1 mM EDTA. Cells were washed twice with PBS and viable cells were counted with a Trypan Blue solution. T cells were negatively purified according to an EasySep^TM^ Human T cell Enrichment kit protocol. Viable T cells were counted with Trypan blue and resuspended in complete RPMI medium, i.e. RPMI 1640 GlutaMAX™, 10% FCS, 100 units/ml penicillin and 100 μg/ml streptomycin. 2,000,000 cells/tube were cultured overnight at 37°C and treated with PMA (10 ng/ml) and Ionomycin (1 μM) in presence of MG-132 (5 μM) for different time points.

### NF-κB reporter gene assays

An NF-κB reporter (Luc)—HEK293 cell line was generated by lentiviral infection using the Cignal Lenti NF-κB Reporter Luc system (SA Biosciences CLS-013L-8,) and was cultured in DMEM/GlutaMAX™ (Gibco) containing 4.5 g/L D-Glucose and 25 mM HEPES, supplemented with 10% heat-inactivated fetal calf serum (PAA). For cell passaging, the complete medium was supplemented with 1 μg/ml Puromycin (Sigma). One day before the experiment, the medium was changed for fresh medium without antibiotic. Transfection was performed in sterile Costar Corning^®^ 96 well white flat bottom polystyrene TC-treated microplates using cell suspensions of 30’000 cells/ well using 0.1 μg total plasmid DNA and 0.3 μl X-tremeGENE™ 9 DNA transfection reagent (Roche). Transfected cells were incubated overnight at 37°C before lysis into Britelite reagent (PerkinElmer^®^). Luciferase activity was analyzed using an EnVision Multilabel Reader.

### SDS-PAGE and western blot procedures

Lysates were resolved using 4–12% Bis-Tris gradient 1.5 mm x 10 well-SDS gels (NuPAGE^®^, Thermo Fisher) in MES buffer in the presence of antioxidant (Thermo Fisher). Electro-transfer of proteins was carried out using the dry iBlot^®^ system 1 (Thermo Fisher) and PVDF iBlot^®^ gel transfer stacks. Blotted membranes were blocked with LI-COR^®^ Blocking solution diluted with PBS 1:1 for one hour at room temperature. Membranes were incubated with primary antibodies overnight at 4°C and subsequently incubated with dye coupled secondary antibodies: Either a goat anti-Rabbit IgG conjugated to IRDye800CW (LI-COR^®^) or a F(ab')2-Goat anti-Mouse IgG (H+L) conjugated to Alexa Fluor^®^ 680 (Thermo Fisher). All antibodies were diluted with 0.1% Tween 20 in [1:1 LI-COR® blocking / PBS]. The primary antibodies were used at 1/1000, the mouse secondary antibody at 1/5000 and the rabbit secondary antibody at 1/10000. Membranes were analyzed on a LI-COR® Odyssey Scanner using 700 nm and/or 800 nm channels according to the manufacturer’s manual. Densitometry analyses were carried out using the ImageJ software.

## Results

### Co-expression of CARD11, BCL10 and MALT1 leads to MALT1 post-translational modifications

There is long standing evidence that MALT1 readily associates with BCL10 [24, 25]. Furthermore, several reports have shown that overexpression of BCL10 can activate MALT1[9, 10]. However, MALT1 is not active in lymphocytes unless the T- or B-cell receptors are stimulated with antigens, inferring that, under physiological conditions, the MALT1-BCL10 interaction may be permissive only. In fact, the CARD11 protein was identified as a third essential component to assemble a tripartite CARD11-BCL10-MALT1 (CBM) complex into lipid rafts at the immunological synapse, following TCR stimulation [26, 27].

**Fig 2:**
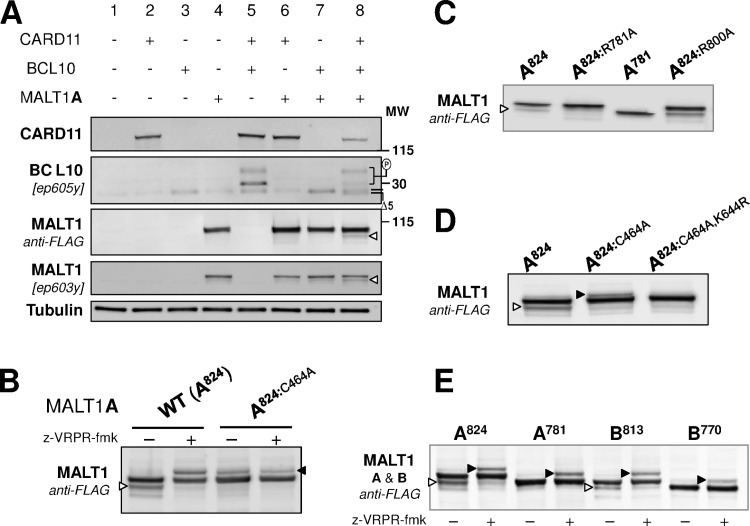
Ectopic CBM reconstitution triggers MALT1 auto-cleavage and ubiquitination. **(A)** Constituents of the CBM complex ─ CARD11-L244P, BCL10 and MALT1 (N-terminal FLAG) ─ were ectopically expressed in HEK293 cells individually or in combination (CBM reconstitution assay). 24h after transfection, lysates were analyzed with anti-CARD11, anti-BCL10 (ep605y), anti-MALT1 (ep603y) and anti-FLAG antibodies. BCL10 cleaved by MALT1 is indicated with Δ5. Phosphorylated BCL10 species are indicated with (P). The white arrow head points to a faster migrating species of MALT1. **(B)** CBM reconstitution was performed in the presence of z-VRPR-fmk, a covalent peptidic inhibitor of MALT1 protease (10) or by using a catalytically deficient MALT1-C464A mutant form. The white arrow head points to a faster migrating species of MALT1, the black one to a slower running species. **(C)** MALT1A-824 (WT), R781A, 1–781 and R800A expression constructs were submitted to a CBM reconstitution assay and analyzed by Western Blot with an anti-FLAG antibody to detect MALT1. This identified the main self-cleavage product (white arrow) to result from cleavage at R781. **(D)** MALT1A-824 WT, C464A and C464A/K644R expression constructs were submitted to a CBM reconstitution assay and analyzed by Western Blot with an anti-FLAG antibody to detect MALT1. This showed that the slower migrating species of MALT1 in our experiments (black arrow) corresponds to the K644-monoubiquitinated MALT1 species identified by Thome and coll. [19]. **(E)** MALT1A and MALT1B both undergo C-terminal cleavage and ubiquitination. CBM reconstitution was performed with MALT1A-824, R781-cleaved MALT1A (MALT1A-781), MALT1B-813 and R770-cleaved MALT1B (MALT1B-770), in the absence or presence of z-VRPR-fmk. The white arrow head points to faster migrating species of MALT1A and B, the black one to a slower running species of these two isoforms.

Here, we reconstituted the CBM complex by transient transfection in HEK293 cells (thereafter referred to as CBM reconstitution assay), in conditions relying on CARD11 to trigger MALT1 proteolytic activity. For this purpose, we used a previously described activated mutant form of the CARD11 protein (L244P) [28]. In a co-transfection experiment together with BCL10 and MALT1 isoform A (MALT1A), the requirement for each CBM component was analysed. The levels of expressed BCL10 remained strikingly low unless CARD11-L244P was co-expressed (Fig 2A, lane 3 and 5), thus suggesting stabilization of BCL10 by CARD11. In fact, this observation is consistent with published studies showing that CARD11 induces phosphorylation and stabilization of BCL10 [29, 30]. Co-expression of MALT1 in the absence of CARD11 only slightly enhanced BCL10 levels (Fig 2A, lane 3 and 7). The protease activity of MALT1 became detectable when all three components of the CBM complex were co-expressed, as shown by cleavage of BCL10 and disappearance of phospho-BCL10 species (Fig 2A, lane 8; S1 Fig). In addition to BCL10 cleavage, reconstitution of the active CBM complex produced a faster migrating species of MALT1 (Fig 2A, lane 8). This band was barely detectable in the absence of either BCL10 or CARD11-L244P (Fig 2A, lanes 6 and 7), thereby indicating that this modification of MALT1 is indeed dependent on CBM-complex formation.

To obtain further evidence, we used the active site covalent MALT1 protease inhibitor z-VRPR-fmk [10] as well as a protease-deficient MALT1-C464A mutant protein [24]. Treatment with z-VRPR-fmk led to disappearance of the MALT1 faster migrating species (Fig 2B). This faster migrating species was also not detected with MALT1-C464A (Fig 2B). These observations suggested that the faster migrating species likely represents a self-cleavage product of MALT1A. Remarkably, an additional and slower migrating species of MALT1A (Fig 2B) became detectable upon treatment of WT-MALT1 with z-VRPR-fmk or when MALT1-C464 was expressed. This suggested that the underlying modification of MALT1 is negatively regulated by the protease function of MALT1.

Taken together, these results indicate that simultaneous ectopic expression of activated CARD11, BCL10 and MALT1 results in post-translational modifications of MALT1.In fact, similar findings were obtained upon expression of CARD9 instead of CARD11 (S2 Fig). CARD9 is a short paralogue of CARD11 expressed by myeloid cells, previously shown to drive Dectin-dependent NF-κB signaling in e.g., dentritic cells, in a MALT1 protease-dependent manner [31].

### MALT1 catalyzes its own cleavage after residue R781

The above observations suggested that the faster migrating species of MALT1 most likely represents an auto-proteolysis product. In addition, given its relatively small size difference compared to full length MALT1 and its detection by an anti-N terminal antibody, the cleavage would be expected to occur close to the C-terminal end of the protein.

Analyses of MALT1 cleavage determinants using positional scanning libraries [32] and knowledge obtained from its known substrates [3] have suggested a minimal SR consensus motif preceding cleavage sites. In fact, MALT1 contains only two such SR motifs close to the C-terminus: R781 and R800. To determine the site of MALT1 self-cleavage, we submitted HEK293 cells to the CBM-reconstitution assay using either, MALT1 WT, R781A or R800A expression constructs. While the MALT1 WT and the R800A mutant proteins were cleaved, the MALT1-R781A protein resisted proteolysis and a MALT1 species encompassing residues 1–781 migrated to the same level as the cleaved MALT1 fragment (Fig 2C). Therefore, these data identified R781 as a novel MALT1 auto-cleavage site. This site appears well conserved across species (S2 Table).

### MALT1 protease deficiency leads to increased mono-ubiquitination

Several post-translational modifications of MALT1 have been reported [33]. In particular, a mono-ubiquitinated form of MALT1 was recently described and K644 was identified as the ubiquitinated residue [19]. To elucidate if this modification accounts for the slower migrating species of MALT1 observed in our experiments, MALT1 WT, MALT1-C464A and MALT1-C464A-K644R were submitted to the CBM reconstitution assay in HEK293 cells. In contrast to MALT1-C464A, no slower migrating MALT1 protein species was found associated with MALT1-C464A-K644R, thereby confirming that K644 is the site for this post-translational modification of MALT1 (Fig 2D). Evidence for ubiquitination at this site was further provided by using anti-ubiquitin immunoblotting (S3A and S3B Fig) and by showing sensitivity of the modification to treatment with the ubiquitin ligase USP2 (S3C Fig).

### Both isoforms of MALT1 are modified upon CBM reconstitution

After identification of post-translational modifications associated with MALT1A, we asked if similar modifications would occur in the less characterized MALT1B isoform. For this purpose, we submitted both isoforms in parallel to the CBM reconstitution assay in the absence or presence of z-VRPR-fmk. A pattern similar to that described above for MALT1A was found in MALT1B (Fig 2E). C-terminal self-cleavage of MALT1B at R770 (equivalent of R781 in MALT1A, Fig 1) was confirmed by site-directed mutagenesis (Fig 2E).

### Endogenous MALT1 undergoes stimulation-induced mono-ubiquitination followed by self-cleavage

**Fig 3:**
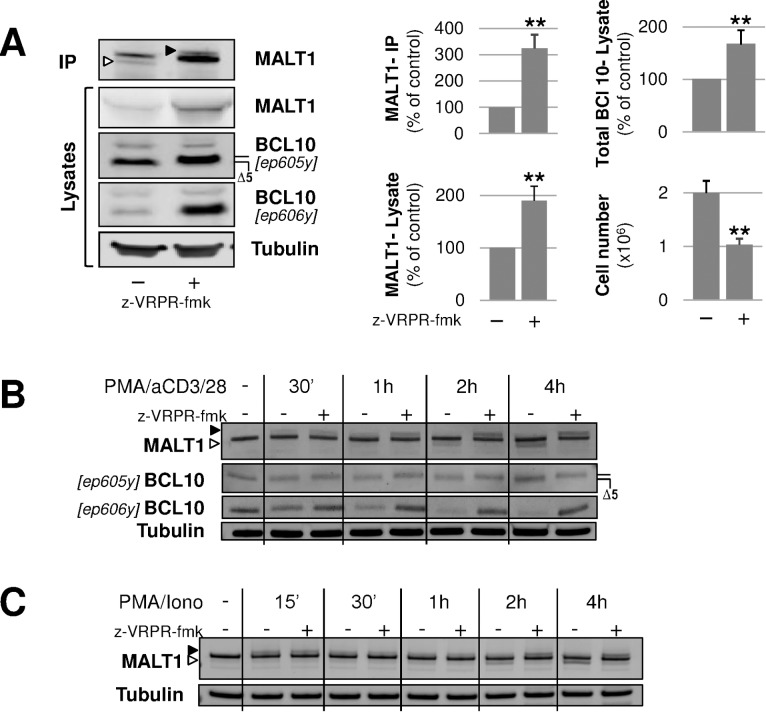
Post-translational modifications of MALT1 in human lymphocytes. **(A)** OCI-Ly3 cells were grown for 4 days in the presence or absence of 50 μM z-VRPR-fmk. Cells were harvested for MALT1 immunoprecipitation and lysate analysis using an anti-MALT1 antibody (H-300), as described in the Methods section. The MALT1 faster and slower migrating species are indicated with white and black arrow heads, respectively. The BCL10 antibody recognizing more specifically the uncleaved form of BCL10 (ep606y) was reported already [38]. Densitometry of MALT1and BCL10 signal intensities was measured in 3 independent experiments and is shown as Mean ± SD. Cells were counted at the end of each experiment and counts are represented as Mean ± SD (N = 3). Statistical significance was calculated using the Student T-test. **(B)** Jurkat cells were stimulated with PMA (10 ng/ml) anti-CD28 (1 μM) and anti-CD3 (1 μM) in the presence of 5 μM MG-132, for various times before full cell extraction and analysis of MALT1 (MT1/410 antibody) and BCL10 (antibodies specified on the figure) by immunoblotting. The MALT1 faster and slower migrating species are indicated with a white and a black arrow head, respectively. **(C)** Primary human CD3 T cells were stimulated with PMA (10 ng/ml) and Ionomycin (1 μM) in the presence of 5 μM MG-132, for various times before full cell extraction and analysis of MALT1 (MT1/410 antibody). The MALT1 faster and slower migrating species are indicated with a white and a black arrow head, respectively. Anti-tubulin immunoblots are provided as loading controls.

To investigate endogenous MALT1 regulation, we first looked into human activated B cell-like diffuse large B-cell lymphoma (ABC-DLBCL) cells, which are characterized by chronic active B-cell receptor signalling [34]. In these lymphoma cells the CBM complex is constitutively assembled, resulting in strong MALT1 protease activity and NF-κB activation [35,36]. In OCI-Ly3 cells, representing a case of ABC-DLBCL, constitutive CBM activation is the consequence of the activating mutation L244P in the coiled-coil domain of CARD11 [28]. In fact, OCI-Ly3 cells represent a setting with endogenous CBM components that matches the transfection paradigm used above in HEK293 cells. In untreated OCI-Ly3 cells, immuno-precipitated MALT1 was detected as a doublet on immunoblots (Fig 3A). Treatment with z-VRPR-fmk led to complete disappearance of the lower protein band and to detection of a slower migrating form, a pattern fully consistent with the CBM reconstitution experiments in HEK293 cells reported above. While prolonged treatment with z-VRPR-fmk reduced proliferation by 2-fold, in line with previous studies [35, 36], it significantly increased the levels of MALT1 and BCL10 proteins (Fig 3A), which suggested that the catalytic function of MALT1 may influence steady-state levels of these CBM components after activation. Further evidence for stabilization of MALT1 and BCL10 when the MALT1 protease is inhibited was obtained by performing the CBM reconstitution assay in the presence of cycloheximide in order to block protein synthesis (S4 Fig).

We then looked at more acute stimulatory conditions using the human leukemic T-cell- Jurkat line. In Jurkat cells, stimulation with PMA/anti-CD28 antibody led to detection of a slower migrating MALT1 species at the earliest time point measurement (30 min) showing progressive decline over time (Fig 3B). This slower running species was stabilized in the presence of z-VRPR-fmk. MALT1 protease activity was measured by following BCL10 cleavage which was marginal after 30 min of stimulation but gradually increased to peak at 2h (Fig 3B). These observations are consistent with the work of Pelzer et al. [19] who showed that mono-ubiquitination of MALT1 occurs early on, shortly before optimal detection of MALT1 proteolytic activity. In addition, MALT1 auto-proteolytic activity was clearly detectable at 2h of stimulation and was increased at 4h, a time when mono-ubiquitinated MALT1 species are no longer detectable (Fig 3B). Therefore, based on steady-state detection levels, MALT1 mono-ubiquitination appears to precede MALT1 auto-proteolysis.

A similar kinetics of events was observed when stimulating human primary CD3 T cells (Fig 3C). Furthermore, a band pattern consistent with cleavage at R781 (R789 in mouse MALT1) and mono-ubiquitination of MALT1 was observed upon PMA/Ionomycin stimulation of T cells isolated from wild-type and catalytically-deficient MALT1 knock-in mice, respectively [37] (S5 Fig).

Altogether, MALT1 mono-ubiquitination and C-terminal self-cleavage do occur with endogenous proteins upon acute or chronic activation of antigen-receptor pathways.

### C-terminal self-cleavage inhibits MALT1 function

**Fig 4:**
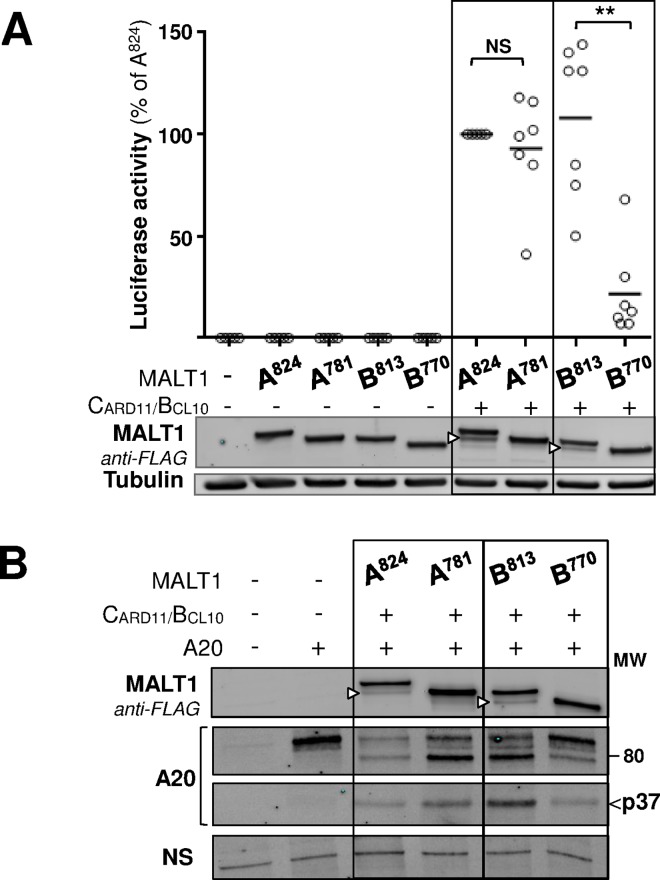
Functional impact of C-terminal cleavage on MALT1 isoforms A and B. **(A)** NF-κB luciferase reporter gene assay in HEK293 cells transfected with either, MALT1A WT, MALT1A-781, MALT1B WT, or MALT1B-770 in the absence or presence of CARD11-L244P. Luciferase activity was recorded after 24h. Data show the mean of triplicate determinations from 7 independent experiments. Statistical significance was calculated using the Student T-test. Western blot analysis, performed in parallel to control for protein expression, is shown below with samples from a representative experiment. An anti-tubulin immunoblot is provided as loading control. **(B)** CBM reconstitution was performed using MALT1A, MALT1B as well as their constitutively cleaved variants, together with the MALT1 protease substrate A20. Immunoblot analyses for MALT1 show the respective C-terminal auto-cleavage bands (white arrow heads). For A20, they show cleaved fragments as previously described [9, 22]. A20p37 is a proteasome sensitive degradation product of A20 [9]. Its weak detection when MALT1A WT is used is consistent with high proteolytic activity resulting in early processing of A20 and disappearance of A20p37 by the time of harvest. A non-specific (NS) band detected by the anti-A20 antibody is provided as loading control.

To explore the functional consequences of MALT1 C-terminal auto-cleavage, MALT1 WT isoforms A and B (A^824^, B^813^) as well as their respective constitutively cleaved mutant forms MALT1A-781 (A^781^) and MALT1B-770 (B^770^) (Fig 1) were compared side by side. First, we used a nuclear factor- kappa B (NF-κB) reporter gene assay in HEK293 to assess the capacity of these MALT1 variants to activate NF-κB upon co-transfection with CARD11-L244P. In this assay, MALT1A and MALT1 B WT proteins as well as the MALT1A-781 mutant protein were active (Fig 4A). In sharp contrast, the ability to stimulate NF-κB was severely impaired in the C-terminally truncated MALT1B-770 mutant protein (Fig 4A).

We then probed the proteolytic function of these MALT1 variants. Each MALT1 construct was co-expressed in HEK293 cells together with CARD11-L244P, BCL10 and the MALT1 proteolytic substrate A20. Cleavage of A20 by MALT1 has been known to generate a complex pattern of fragments, some of which, *e*.*g*., p37, displaying limited stability due to proteasomal degradation [9, 22]. Both MALT1 isoforms displayed proteolytic activity towards A20. Activity of MALT1A was strong under our assay conditions, resulting in disappearance of full length as well as cleaved fragment signals (Fig 4B). Activity of MALT1B was reduced compared to MALT1A, as seen in the overall pattern and intensity of A20 full length and cleaved products (Fig 4B). C-terminal truncation further reduced proteolytic activity in both isoforms resulting in almost complete abrogation in the case of MALT1B, as judged from the very limited cleavage pattern of A20 (Fig 4B). More substantiation of the impact of MALT1 auto-proteolytic activity on its protease function, using cylindromatosis (CYLD) which is another reported MALT1 substrate [11], is provided further below.

As mentioned above, MALT1B misses one key TRAF6 binding motif (T6BM1). Cleavage at R781 (MALT1A)/ R770 (MALT1B) removes another key TRAF6 binding motif (T6BM3). Upon C-terminal auto-cleavage, MALT1B would therefore become devoid of the two major TRAF6 binding sites (T6BM1 and T6BM3) **─** T6BM2 is another TRAF6 binding motif previously reported which appears less critical [39] (Fig 1). The above data revealed similarities between the catalytic potential and the capacity to activate NF-κB, of MALT1 isoforms and their auto-cleavage products, which seemed to match their degree of impairment in TRAF6-binding capability. This prompted us to study if TRAF6 might regulate MALT1 proteolytic function.

### TRAF6 can directly activate MALT1, promoting self-cleavage at R149

**Fig 5:**
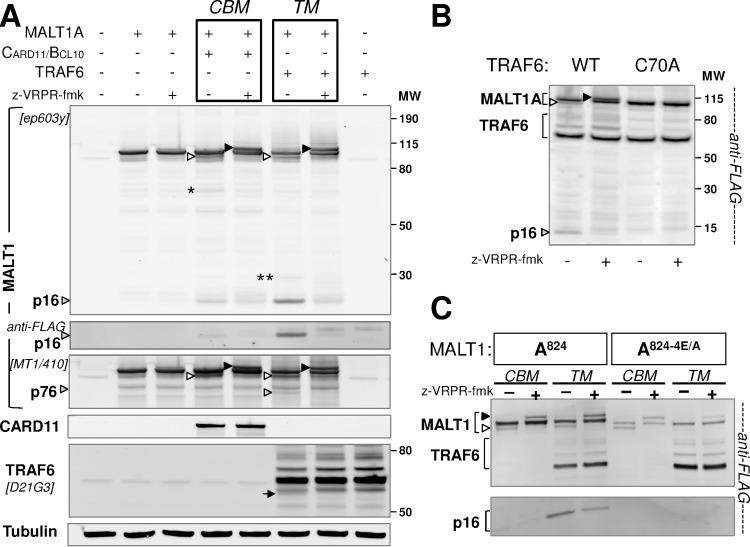
TRAF6 is able to trigger auto-cleavage and mono-ubiquitination of MALT1. **(A)** Comparison of CBM reconstitution assay as in Fig 1 (transfection ratio 1:1:1) and TM reconstitution assay (transfection ratio 1:1:1, using a control plasmid) in HEK293 cells. Immunoblot analyses are shown with anti-MALT1 antibodies (specified on the figure), anti-FLAG antibody to confirm the MALT1 p16 fragment, and anti-CARD11 as well as anti-TRAF6 antibodies. Both assays led to MALT1 C-terminal auto-cleavage (white arrow head) and mono-ubiquitination in the presence of z-VRPR-fmk (black arrow head). N-terminal MALT1 auto-cleavage was significantly detected only in the context of he TM assay, as shown by the generation of the two fragments p76 and p16 (both denoted with a grey arrow head). * and ** denote bands resulting from secondary auto-cleavage reactions, at the C- and N- terminus, respectively. The arrow in the TRAF6 immunoblot points to a possible minor cleavage product of TRAF6 which disappears in the presence of z-VRPR-fmk. **(B)** The TM reconstitution assay was performed using FLAG-TRAF6 WT or the FLAG-TRAF6-C70A mutant construct. Western Blot analysis with anti-FLAG antibody is shown. C-terminal auto-cleavage of MALT1 (white arrow head) and mono-ubiquitinated MALT1 (black arrow head) were observed with TRAF6 WT only. **(C)** Comparison of MALT1 WT and MALT1A-E4/A (TRAF6-binding deficient) in CBM and TM reconstitution assays. Immunoblotting with anti-FLAG antibody is shown. MALT1 C-terminal auto-cleavage is denoted with a white arrow head, N-terminal auto-cleavage (p16) is shown in the bottom panel, and mono-ubiquitination in the presence of z-VRPR-fmk is denoted with a black arrow head.

To evaluate the influence of TRAF6 on MALT1, reconstitution experiments were carried out in the absence of co-transfected BCL10 and CARD11-L244P. Co-expression of MALT1 together with TRAF6 (thereafter referred to as TM reconstitution assay) was sufficient to induce the auto-cleavage/mono-ubiquitination pattern of MALT1 as observed upon CBM reconstitution (Fig 5A). Co-expression of MALT1 with alternative TRAF family members like TRAF2 or TRAF3 failed to induce auto-cleavage of MALT1, indicating specificity for TRAF6 (S6A Fig). Because the E3 ubiquitin ligase function of TRAF2 was reported to require the cofactor sphingosine-1-phosphate [40], we further tested the influence of added sphingosine-1-phosphate or co-expressed sphingosine kinase. However, this did not induce TRAF2-dependent MALT1 auto-proteolysis (data not shown).

Binding of TRAF6 to MALT1/BCL10 activates the E3 ubiquitin ligase function of TRAF6, allowing for poly-ubiquitination of NEMO [33, 41]. To determine if TRAF6 ligase function is involved in TRAF6-dependent activation of MALT1, co-transfections were carried out with the TRAF6-C70A mutant protein which is unable to interact with the Ubc13/E2 ubiquitin ligase complex resulting in loss of TRAF6 E3 ubiquitin ligase activity [42, 43]. TRAF6-C70A failed to induce auto-cleavage of MALT1 (Fig 5B), implying that interaction of TRAF6 with the Ubc13/E2 ubiquitin ligase complex is required to activate MALT1. Lack of MALT1 mono-ubiquitination upon addition of z-VRPR-fmk further suggested an impaired complex formation between MALT1 and TRAF6-C70A. Similarly, the TRAF6-F118A mutant protein which cannot dimerize ─ a mechanism required for TRAF6 function [44] ─ failed to induce auto-cleavage of MALT1 (S6B Fig). By contrast, co-expression of TRAF6-K124R ─ a mutant protein defective in self-ubiquitination, which is believed to sustain TRAF6 activity [45, 46] ─ led to similar auto-cleavage and mono-ubiquitination of MALT1 as observed with TRAF6 WT (S6B Fig). Taken together, the results obtained with these TRAF6 mutant proteins indicate that dimerized and fully functional TRAF6 is required for activation of MALT1 proteolytic function, whereas TRAF6 auto-ubiquitination at K124 is less critical.

Remarkably, another MALT1 self-cleavage event became apparent under the experimental setting of the TM reconstitution assay. This corresponds to N-terminal auto-proteolytic cleavage of MALT1 at R149, recently reported by Baens et al. [18] (Fig 5A, S6B Fig). Cleavage at R149 releases two fragments, p16 and p76 in MALT1A (Fig 1). TM reconstitution was by far more effective than CBM reconstitution at eliciting cleavage of MALT1A at R149, as seen by monitoring the production of both p16 and p76 fragments (Fig 5A). As expected, cleavage at R149 was abolished when the catalytic deficient TRAF6-C70A was used instead of WT TRAF6 in the TM reconstitution assay (Fig 5B).

To seek more evidence for the role of TRAF6 in promoting auto-cleavage of MALT1 at R149, we used a mutant form of MALT1 in which the 4 critical Glutamine residues within the TRAF6 binding motifs (see Fig 1) were changed into Alanine residues. These mutations were previously shown to abrogate TRAF6-binding capability in the resulting mutant protein [39]. The MALT1-4E/A mutant protein was analyzed side by side with MALT1-WT in CBM- and TM-reconstitution assays. In the CBM assay, both MALT1-WT and MALT1- 4E/A were active and able to produce a C-terminally auto-cleaved fragment (Fig 5C). Furthermore, both proteins had similar proteolytic activity towards the MALT1 substrate CYLD when added to the CBM reconstitution assay (S7A Fig). However, cleavage at R149 was completely absent in MALT1-4E/A despite pronounced cleavage at R781, in contrast to MALT1-WT. In a TM reconstitution assay, MALT1-4E/A remained inactive as expected, showing complete absence of auto-proteolysis (Fig 5C) as well as lack of proteolytic activity towards CYLD (S7B Fig). By contrast, MALT1-WT was active in the TM reconstitution assay and could auto-cleave at both R781 and R149 (Fig 5C, S7B Fig).

### Dominant-negative TRAF6 blocks MALT1 self-cleavage at R149 without interfering with CBM-induced MALT1 activation

**Fig 6:**
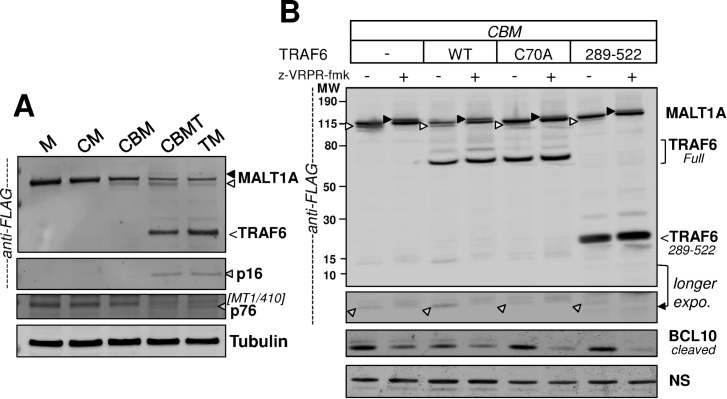
MALT1 auto-cleavage at R149 is induced by TRAF6. **(A)** MALT1A was expressed in HEK293 cells, either alone, or together with CARD11-L244P, or as part of a CBM reconstitution assay (transfection ratio 1:1:1:1 using a control plasmid), or a TM reconstitution assay (TRAF6 + MALT1 (transfection ratio 1:1:2 with “2” referring to a control plasmid), or a CBMT reconstitution assay (transfection ratio 1:1:1:1). Western Blot analyses with anti-FLAG and anti-MALT1 (MT1/410) antibodies are shown, displaying MALT1 auto-cleaved fragments (C-terminal, white arrow head; N-terminal depicted as p16/p76, grey arrow heads). Loading controls with an anti-tubulin antibody are also provided. **(B)** The CBMT reconstitution assay was performed as in (A) using either TRAF6-WT, or TRAF6-C70A, or TRAF6 289–522. Anti-FLAG Western Blot analyses are shown, displaying MALT1 auto-cleaved fragments (C-terminal, white arrow heads; N-terminal p16, grey arrow heads) as well as MALT1 mono-ubiquitinated species (black arrow heads). An anti-cleaved BCL10 immunoblot providing evidence for proteolytic activity of MALT1 is also shown together with a non-specific (NS) band detected by the BCL10 antibody, as loading control.

TRAF6 is known to connect the CBM complex with downstream NF-κB signaling. In order to put the above observations into pathway perspective, we combined the CBM and TM assays (CBMT reconstitution) to ask about the influence of TRAF6 when MALT1 can engage into CBM complexes. Addition of TRAF6 to the CBM reconstitution assay was sufficient to induce production of the p16/p76 fragments resulting from N-terminal auto-cleavage of MALT1A at R149 (Fig 6A). Replacing TRAF6-WT by the catalytically deficient TRAF6-C70A mutant in this assay prevented induction of cleavage at R149A but was without effect on either CBM-induced auto-cleavage at R781 or CBM-induced BCL10 cleavage, or CBM-induced MALT1 mono-ubiquitination in the presence of z-VRPR-fmk (Fig 6B).

To strengthen these observations we used TRAF6 289–522, a validated inactive and dominant-negative mutant form of TRAF6 that lacks the complete zinc-binding region [21]. Similar to TRAF6-C70A, this mutant abrogated induction of MALT1A auto-cleavage at R149 but had no effect on the CBM-induced modifications in MALT1A or on CBM-induced proteolytic cleavage of BCL10 (Fig 6B).

Altogether, these experiments demonstrated that TRAF6 conveys a specific input onto the CBM to induce self-cleavage of MALT1 at R149.

### TRAF6 triggers auto-proteolysis-dependent MALT1 down-regulation

**Fig 7:**
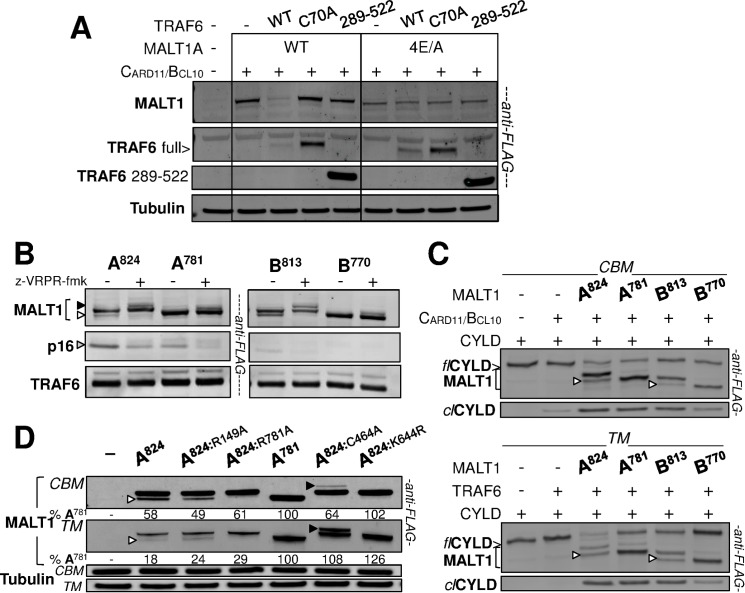
TRAF6 induces auto-proteolysis-dependent down-regulation of MALT1. **(A)** The CBMT reconstitution assay in HEK293 was performed with either MALT1A-WT, or the TRAF6-binding deficient MALT1A-4E/A mutant construct, in the presence of TRAF6-WT, TRAF6-C70A or TRAF6 289–522. Immunoblotting with anti-FLAG antibody is shown. An anti-tubulin immunoblot is provided as loading control. **(B)** The TM reconstitution assay in HEK293 cells was performed using MALT1A, MALT1B as well as their C-terminal truncated variants. Anti-FLAG Western Blot analyses show the respective C-terminal auto-cleavage bands (white arrow head), the respective N-terminal auto-cleavage bands (grey arrow head, p16) as well as the respective mono-ubiquitinated species detected in the presence of z-VRPR-fmk (black arrow head). They also provide TRAF6 co-expression levels (bottom panels). **(C)** CBM and TM reconstitution assays in HEK293 cells were performed in the presence of co-expressed CYLD, using MALT1A, MALT1B, as well as their C-terminal truncated variants. Anti-FLAG Western Blot analyses show MALT1 C-terminal auto-cleavage bands (white arrow heads) as well as CYLD full length (*fl*) and cleaved fragment (*cl*) levels. Anti-CYLD immunoblots are provided in S7C Fig. **(D)** CBM and TM reconstitution assays in HEK293 cells were performed using MALT1A-WT, several point mutant forms thereof: R149A, R781A, C464A, K644R, as well as the MALT1A-781 truncated form mimicking constitutive C-terminal auto-cleavage. Immunoblots with anti-FLAG antibody are shown. Densitometry analysis of MALT1 signals as percentage of the signal obtained with MALT1A 1–781 is provided underneath. MALT1 C-terminal auto-cleavage is denoted with a white arrow head and mono-ubiquitination with a black arrow head. An anti-tubulin immunoblot provides loading controls for the both assays.

Looking closely at immunoblots from TM and CBMT reconstitution assays (e.g. Fig 5 and Fig 6), it seemed that the overall signals for MALT1 were lower as compared to CBM reconstitution assays. This led us hypothesize that TRAF6 might have an impact on MALT1 stability through induction of MALT1 auto-proteolysis. To strengthen the rationale for this hypothesis, we repeated the CBMT reconstitution assay and compared MALT1-WT and MALT1-4E/A. The levels of MALT1-WT protein observed under the CBM assay condition were drastically reduced under the CBMT condition if TRAF6-WT was used (Fig 7A). Instead, they remained similar to those of the CBM assay condition if TRAF6-C70A or TRAF6-289-522 were used (Fig 7A). In sharp contrast, protein levels of MALT1-4E/A remained similar in all assay conditions, as expected because this mutant cannot bind TRAF6 (Fig 7A). This data therefore confirmed that TRAF6 does bring in a signal to diminish MALT1-WT expression levels.

To test the hypothesis of auto-cleavage-driven down-regulation of MALT1 protein levels, we submitted the two MALT1 isoforms and their respective C-terminally truncated versions to a TM assay and monitored the extent of their TRAF6-induced auto-cleavage at R149. Cleavage of MALT1A at R149, monitored by observation of the p16 N-terminally cleaved fragment, was prominent and correlated with reduced levels of full-length MALT1A that could be stabilized in the presence of z-VRPR-fmk. C-terminal truncation of MALT1A significantly reduced its capacity for self-cleavage at R149 (Fig 7B, left). The weakened ability of MALT1B for self-cleavage at R149 as compared to MALT1A was further diminished after C-terminal cleavage (Fig 7B, right). Of note, for MALT1A-781, MALT1B and MALT1B-770, there was no significant protein degradation as judged from the lack of impact of z-VRPR-fmk, in contrast to MALT1A. This experiment therefore indicated that reduced MALT1 protein levels correlate with the capacity of MALT1 to cleave at R149, which itself results from its capacity to interact with TRAF6.

We then tested if increased self-cleavage at R149 in the presence of TRAF6 would reflect an overall increase in proteolytic activity of MALT1. For this purpose, we submitted MALT1A, MALT1B and their C-terminally cleaved versions to CBM and TM reconstitution assays in the presence of CYLD. Extent of CYLD cleavage in the CBM assay confirmed the lower proteolytic activity of C-terminally truncated MALT1, very pronounced for MALT1B (B770) as well as the reduced activity of MALT1B vs. MALT1A (Fig 7C), as observed with A20 (Fig 4B). These differences in activity became more prominent in the TM assay, indicating that C-terminal self-cleavage does hamper proteolytic function of MALT1 triggered by TRAF6 (Fig 7C). Correlating with this observation, TRAF6 strongly reduced MALT1A protein levels as compared to the CBM assay levels. TRAF6 had a more limited impact on MALT1B levels and, remarkably, had no effect at all on C-terminally truncated MALT1A and MALT1B levels (Fig 7C).

In contrast to MALT1 WT, the catalytically-compromised MALT1-K644R and -C464A mutant proteins appeared stable when analyzed in a TM reconstitution assay as their protein expression levels were similar to those of the CBM assay conditions (Fig 7D). This further indicated that proteolytic competency is required for TRAF6-mediated MALT1 down-regulation. In addition, the non-cleavable auto-cleavage site mutants MALT1-R149A and MALT1-R781A were strongly down-regulated in the TM assay as compared to the CBM assay, like MALT1-WT (Fig 7D).

Altogether, these experiments revealed that down-regulation by TRAF6 of MALT1 expression levels requires MALT1 proteolytic function and its ability to interact with TRAF6. The extent of down-regulation appears to correlate with the capacity of MALT1 to cleave CYLD and to undergo TRAF6-mediated self-cleavage at R149. However, auto-cleavage at either R149 or R781 is unlikely to be rate-limiting for MALT1 turnover.

### The enhanced signaling capacity of N-terminally auto-cleaved MALT1 is lost after C-terminal auto-cleavage

**Fig 8:**
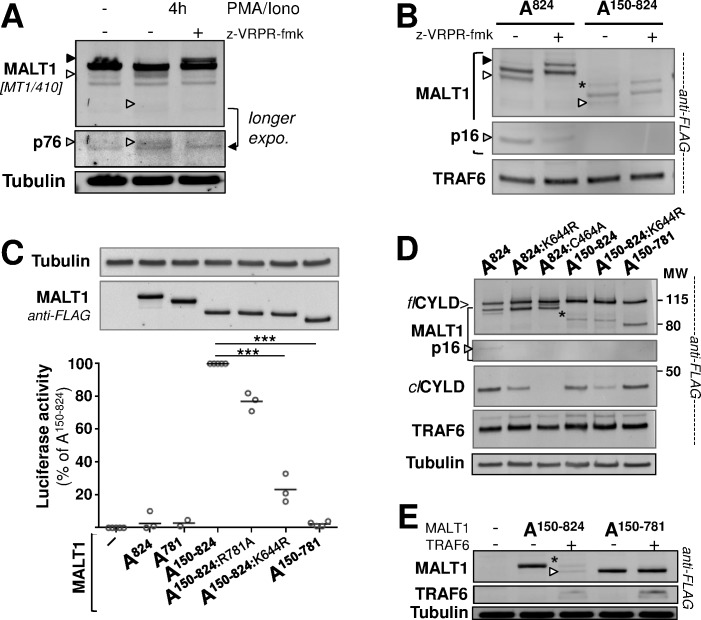
N-terminally auto-cleaved MALT1 requires an intact C-terminus for function. **(A)** Jurkat cells were stimulated with PMA (10 ng/ml) /Ionomycin (1 μM) for 4h, in the presence of 5 μM MG-132 and ± 100 μM z-VRPR-fmk, before full cell extraction and analysis of MALT1 by immunoblotting. The MALT1 faster and slower migrating species are indicated with a white and a black arrow head, respectively. A band corresponding to N-terminally cleaved MALT1 is also indicated with a grey arrow head. **(B)** TM reconstitution assay in HEK293 cells performed with MALT1A WT and MALT1A 150–824. Immunoblot analyses with anti-FLAG antibody are shown. MALT1 C-terminal auto-cleavage is denoted with a white arrow head, N-terminal auto-cleavage with a grey arrow head (p16) and mono-ubiquitination with a black arrow head. TRAF6 co-expression levels are provided in the bottom panel. **(C)** NF-κB luciferase reporter gene assay in HEK293 transfected with MALT1A and variants thereof as indicated, in the absence of CARD11-L244P. Luciferase activity was recorded after 24h. Data show the mean of triplicate determinations from at least 3 independent experiments. Statistical significance was calculated using the Student T-test. Western blot analysis, performed in parallel to control for protein expression, is shown below with samples from a representative experiment. An anti-tubulin immunoblot is provided as loading control. **(D)** TM reconstitution assay in HEK293 cells was performed in the presence of co-expressed CYLD, using MALT1A and variants thereof, as indicated. Anti-FLAG Western Blot analyses show MALT1 expression pattern, in particular the N-terminally auto-cleaved fragment, observable only with MALT1A-WT (grey arrow head). They also display CYLD full length (*fl*) and cleaved fragment (*cl*) levels, as well as TRAF6 co-expression levels. An anti-tubulin blot is provided as loading control (bottom panel). **(E)** Reconstitution assay in HEK293 cells showing MALT1A 150–824 and MALT1A 150–781 expression levels in the absence or presence of co-expressed TRAF6. Immunoblot analysis with anti-FLAG antibody is shown. MALT1 C-terminal auto-cleavage is denoted with a white arrow head. * denotes a slower migrating species of MALT1A 150–824 observed to variable extents upon co-expression with TRAF6 (**B**, **D**, **E**). It is not due to mono-ubiquitination at K644.

MALT1 self-cleavage at R781 or R149 was detectable in Jurkat T cells after PMA/Ionomycin in the presence of the proteasome inhibitor MG-132. However, cleavage at R781 was dominant under these conditions (Fig 8A). Cleavage at R781 removes the key C-terminal T6BM, which is known to loosen binding to TRAF6 and thereby would not favor further self-cleavage at R149. However, it does not prevent it (Fig 7B). Cleavage at R149 removes the death domain in MALT1 and thereby precludes further interaction with BCL10 [47]. However, in the presence of TRAF6, N-terminally cleaved MALT1A was able to generate an R781-cleaved fragment (Fig 8B). Therefore, even if very low levels of double cleaved MALT1 might be generated endogenously following activation ─ double-cleaved endogenous MALT1 species remained undetectable in our experiments, *e*.*g*., in stimulated Jurkat cells ─ we asked how C-terminal truncation of N-terminally cleaved MALT1 might affect its function. Cleavage at R149 was previously shown to be required for MALT1 to control expression of NF-κB target genes in Jurkat T cells [18]. We confirmed that R149-cleaved MALT1A is readily active on NF-κB upon transfection of a HEK293-NF-κB reporter line (Fig 8C). However, MALT1A 150–781 was inactive, providing further evidence for the negative impact of cleavage at R781 on MALT1 function (Fig 8C). In addition, activity associated with MALT1A 150–824 was significantly reduced when K644 was mutated, suggesting that N-terminally-cleaved MALT1 requires mono-ubiquitination for function (Fig 8C).

When co-expressed together with TRAF6, MALT1A 150–824 displayed proteolytic activity towards CYLD (Fig 8D). Proteolytic potential was preserved in MALT1A 150–781 (Fig 8D), thus clearly contrasting with NF-κB activation potential. Like in full length MALT1A, preventing mono-ubiquitination at K644 in MALT1A 150–824 significantly reduced proteolysis of CYLD (Fig 8D). Expression levels of MALT1A 150–824 were drastically reduced upon co-expression with TRAF6 whereas MALT1A 150–781 levels remained insensitive (Fig 8E). These observations are consistent with those reported above for MALT1A-824 and MALT1A-781 (Fig 7), further establishing the direct role of TRAF6 in triggering MALT1 down-regulation.

### Mechanistic insights into MALT1 self-cleavage reactions and mono-ubiquitination

**Fig 9:**
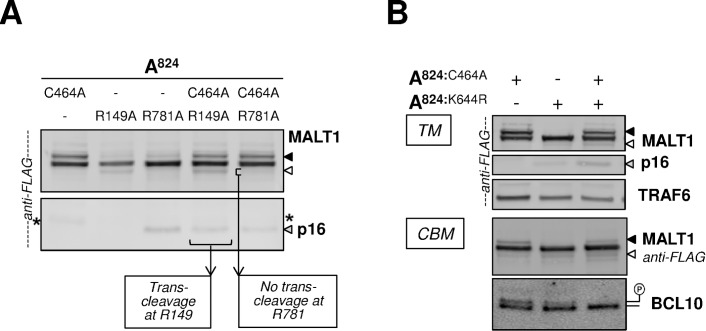
MALT1 auto-proteolysis and mono-ubiquitination mechanisms. **(A)** The TM reconstitution assay performed with MALT1A-C464A, MALT1A-R149A, MALT1A-R781A and combinations thereof. Immunoblotting with anti-FLAG antibody is shown. MALT1 C-terminal auto-cleavage is denoted with a white arrow head, N-terminal auto-cleavage with a grey arrow head (p16) and mono-ubiquitination of MALT1A-C464A with a black arrow head. * denotes a faint band that migrates above p16, which we observed in lanes loaded with MALT1A-C464A samples. **(B)** TM and CBM reconstitution assays were performed with MALT1A-C464A, MALT1A-K644R and the combination of the two. Immunoblots with anti-FLAG antibody are shown. MALT1 C-terminal auto-cleavage is denoted with white arrow heads, N-terminal auto-cleavage with a grey arrow head (p16) and mono-ubiquitination of MALT1A C464A with black arrow heads. The immunoblot with anti-BCL10 antibody (ep605y) shows reduced phosphorylated BCL10 when MALT1A-C464A and MALT1A-K644R are co-expressed, indicative of MALT1 protease activity (see Fig 2A).

Because MALT1 needs to oligomerize to become activated, auto-cleavage reactions may occur either in cis, *i*.*e*., using an intra-molecular mechanism, or in trans, *i*.*e*., on alternative MALT1 molecules within the oligomer. To test these possibilities, we co-transfected MALT1A mutant forms resisting auto-cleavage at either R149 or R781, together with catalytically inactive MALT1A-C464A. Co-expression of MALT1A-R149A together with MALT1A-C464A led to the formation of a p16 fragment released from the C464A-MALT1 protein, confirming that cleavage at R149 can occur in trans (Fig 9A) [18]. By contrast, co-expression of MALT1A-R781A together with MALT1A-C464A did not allow for cleavage of MALT1A-C464A at R781 (Fig 9A), implying that auto-cleavage of MALT1A at R781 follows an intramolecular mechanism.

In our experiments, the maximal level of mono-ubiquitinated MALT1 never exceeded 50% of the total amount of MALT1 protein, suggesting that the mechanism involved might allow for a maximum of one ubiquitin moiety per MALT1 dimer. To test if one ubiquitin per dimer would be sufficient to induce MALT1 protease function we co-expressed the catalytic deficient MALT1A-C464A mutant form together with mono-ubiquitin-deficient MALT1A-K644R. This co-expression induced MALT1A auto-cleavage at R149 in a reconstitution assay with TRAF6, and it reduced phospho-BCL10 species in a CBM reconstitution assay (Fig 9B). Therefore, ubiquitination of MALT1A-C464 appears to allow for proteolytic activation, in trans, of the otherwise barely active MALT1A-K644R protein.

The mechanism for MALT1 mono-ubiquitination remains ill-defined. Binding of MALT1 to BCL10 does not appear to be required because ectopically expressed MALT1A 150–824 remained dependent on an intact K644 to stimulate NF-κB, implying that this MALT1 fragment can be mono-ubiquitinated like full-length MALT1. TRAF6-mediated poly-ubiquitination of MALT1was previously shown to be an important step of the NF-κB signaling cascade by bridging the CBM and the IKK protein complexes [33]. The present work has shown that binding of MALT1 to TRAF6 is not required for MALT1 mono-ubiquitination because the MALT1-4E/A mutant protein was readily modified when co-expressed with activated CARD11 and BCL10 (Fig 5C). Further evidence against this hypothesis came from co-expression studies where defective TRAF6 species like TRAF6-C70A or dominant-negative TRAF6 289–522 hampered N-terminal auto-cleavage, but not mono-ubiquitination of MALT1 (Fig 6B). In fact, dimerization of MALT1 was previously shown to be a pre-requisite for mono-ubiquitination [48] and appears to be the driving mechanism. Together this indicates that a scaffolding context allowing for MALT1 oligomerization, whether through CBM complex assembly, or via binding to TRAF6, may poise MALT1 for mono-ubiquitination.

Among the known MALT1 substrates, HOIL-1, A20 and CYLD are regulators of ubiquitination mechanisms. HOIP is the catalytic subunit of the linear ubiquitin assembly complex and binder of HOIL-1. We tested a catalytically deficient HOIP-C885A mutant protein: It did not inhibit mono-ubiquitination of MALT1 triggered by reconstitution with TRAF6 (S8A Fig). Expression of the de-ubiquitin enzymes A20 or CYLD was also without effect on co-expressed mono-ubiquitinated MALT1-C464A (S8A Fig). Lastly, we tested the hypothesis that MALT1 might be able to remove its ubiquitin chains by taking advantage of its arginine-directed proteolytic activity. In fact, immediately upstream the C-terminal Gly doublet ubiquitin contains two Arg residues, within a motif that bears homology to that recognized by MALT1 in its proteolytic substrates: LVLRLRGGC. We therefore mutated the two Arg residues into Alanines and asked if this would lead to increased ubiquitination in MALT1A WT in the absence of z-VRPR-fmk. This was not the case (S8B Fig).

## Discussion

In this work a novel, prominent, C-terminal auto-cleavage mechanism of MALT1 has been discovered. The auto-proteolytic site was identified after R781 in MALT1 isoform A (R770 in isoform B) and the functional consequences in both isoforms were characterized. Cleavage at this site removes the last 43 amino-acids residues that contain one key TRAF6-binding motif (T6BM3, Fig 1, Fig 2). C-terminal auto-cleavage had limited impact in MALT1A, both in terms of proteolytic function and NF-κB activation potential (Fig 4). By contrast, C-terminal auto-cleavage at R770 had severe consequences in MALT1B. This isoform constitutively lacks another key TRAF6-binding motif (T6BM1, Fig 1). Removal of its C-terminus almost abrogated proteolytic function and NF-κB activation (Fig 4). This result is consistent with recent work showing that mutation of the C-terminal TRAF6 site (T6BM3) in MALT1B completely abrogates NF-κB signaling upon T cell stimulation [20]. It is also consistent with API2-MALT1 fusion proteins like AT7M8, which lacks T6BM1. AT7M8 was competent to activate NF-κB but this was completely suppressed in AT7M8-ΔC80, a C-terminally truncated version lacking T6BM3 [18, 49]. Impaired TRAF6-binding capability of AT7M8-ΔC80 and MALT1B-770 is however unlikely to fully account for their dominant negative-like properties because a MALT1 mutant protein with disabled TRAF6 binding sites (MALT1-4E/A) was still proteolysis competent (Fig 5C and S7A Fig).

Control of MALT1 auto-proteolytic processing by TRAF6 is another key finding of this work. Alternative TRAF family members like TRAF2 and TRAF3 had no effect on MALT1 (S6A Fig). In fact, TRAF2 and TRAF3 do not participate in CBM-driven NF-κB cascades whereas TRAF6 is a key intermediate for connecting the CBM to the IKK complex [33]. Induction of MALT1 activity required the E3 ligase of TRAF6 (Fig 5B) and its capacity to form oligomers (TRAF6 F118A mutant data in S6 Fig). In their pioneering in vitro pathway reconstitution study, Sun et al. demonstrated that MALT1 oligomerization is required to activate TRAF6 ubiquitin ligase [33]. Our data suggest a dual regulation mechanism whereby TRAF6, in turn, affects MALT1 proteolytic processing. An intriguing question is whether TRAF6 might be able to bypass CBM formation in order to activate MALT1. HEK293 cells endogenously express CARD10, a protein similar to CARD11. CARD10 was previously shown to rescue NF-κB activity in a Jurkat cell model of CARD11 deficiency [50]. Therefore, the hypothesis of CBM-independency of TRAF6-mediated activation of MALT1 would need to be thoroughly tested in a complete CARD-deficient background.

Binding to TRAF6 is not a pre-requisite for MALT1 proteolytic function because the MALT1-4E/A mutant protein could be activated through CBM-reconstitution to similar levels as MALT1 WT, as seen in self-cleavage at R781 (Fig 5C) and cleavage of CYLD (S7A Fig). However, several lines of evidence have shown that TRAF6 controls self-cleavage of MALT1 after R149, a site that was identified previously (18). First, reconstitution assays with TRAF6, but not with activated CARD11 and BCL10, allowed for robust MALT1 self-cleavage after R149, as demonstrated by the production of p16 and p76 migration products in Western blot analyses. (Fig 5, Fig 6). Second, addition of TRAF6 WT to CBM-reconstitution assays was sufficient to induce p16/p76 production (Fig 6A) whereas the catalysis deficient TRAF6-C70A mutant protein and the truncated dominant negative TRAF6 289–522 mutant protein both precluded p16 production (Fig 6B). Third, MALT1B or C-terminally truncated MALT1A, which both miss a distinct but important binding site for interaction with TRAF6, T6BM1 and T6BM3 respectively, displayed significantly reduced ability to generate the p16 peptide (Fig 7B) ─ The ratio of self-cleavages at R149 *vs*. R781/770 was actually inverted in MALT1B as compared to MALT1A. Finally, the TRAF6 binding deficient MALT1-4E/A mutant protein which, as described above is catalytically competent and can auto-cleave at R781, was unable to produce p16 under any experimental condition (Fig 5C). On a molecular level, it appears as if TRAF6 would alter the molecular structure of the MALT1 activating complex. When incorporated in the CBM, MALT1 is auto-processed at the C-terminus, indicating that the MALT1 C-terminus and its active site are brought into close proximity. By contrast, TRAF6 seems to stabilize an oligomeric state where the N-terminus of one MALT1 molecule is brought into close proximity of another, allowing trans-cleavage (Fig 9). Supporting these hypotheses, secondary cleavage fragments of MALT1 could be observed at the C-terminus when MALT1 was reconstituted with CARD11-L244P and BCL10 but at the N-terminus in the presence of TRAF6 (Fig 5A, * and **, respectively).

Monitoring MALT1 self-cleavage in T cells showed that C-terminal cleavage becomes detectable after 30 minutes of stimulation, similarly to proteolysis of BCL10 (Fig 3B and 3C). The self-cleavage fragment continued to accumulate over time in the presence of the proteasome inhibitor MG-132. By contrast, N-terminally cleaved MALT1 remained barely detectable (Fig 8A). This observation is consistent with the work of Baens et al [18] who detected the N-terminally cleaved MALT1 fragment using prolonged exposures of their immunoblots. Prominence of C-terminal cleavage over N-terminal cleavage might reflect, on the one hand, a rate-limiting process for the latter. Indeed, recruitment of TRAF6 by the CBM complex could be a mechanism to ensure that only those MALT1 molecules that interact with TRAF6 will be processed at the N-terminus and then released from the CBM. On the other hand, TRAF6 might trigger down-regulation of N-terminally cleaved MALT1, as suggested by our work (Fig 8E), thereby directly impacting detectability of this fragment. Clearly, further work will be required to get a better understanding of self-cleavage regulation mechanisms for endogenous MALT1.

This report has confirmed that N-terminally cleaved MALT1 (MALT1 150–824) can readily activate NF-κB, without requirement for co-expressed CARD11-L244P (Fig 8C) [18]. In addition we have shown that K644, the reported site for mono-ubiquitination of MALT1[19], is essential for both NF-κB activation and proteolytic activity of N-terminally cleaved MALT1 (Fig 8C and 8D). We also characterized MALT1 150–781, i.e. the MALT1 fragment mimicking the product of the two self-cleavage reactions, although our observations with endogenous MALT1 cleavage products (Fig 8A) suggest a low likelihood that double-cleaved MALT1 species can build up to a significant extent. This fragment was still capable of proteolytic cleavage of CYLD (Fig 8D), which is consistent with previous mapping of the sequence determinants for MALT1 proteolytic function [32]. However, NF-κB activation potential of MALT1 150–781 was abrogated, suggesting that TRAF6 is responsible for enhanced signaling through its interaction with MALT1 150–824, which would be hindered following C-terminal cleavage.

**Fig 10:**
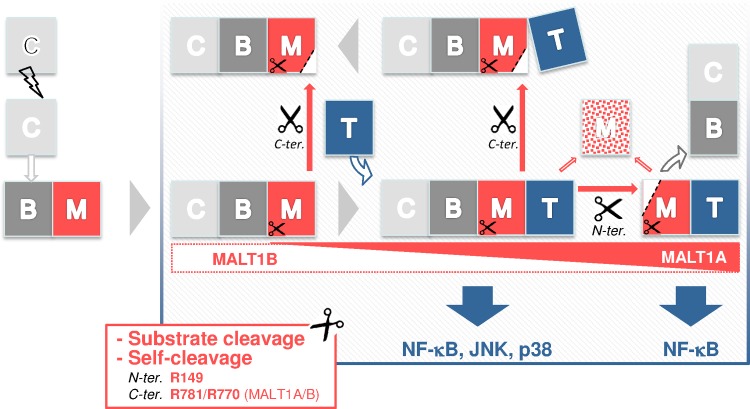
Influence of TRAF6 on MALT1 function, scaffolding, self-cleavage reactions and protein turnover. The model represents the capacity of MALT1 isoforms (M) to interact with their scaffolding partners CARD11 (C), BCL10 (B) and TRAF6 (T) and how self-cleavage reactions impact this capacity. Upon CARD11 activation resulting in CBM assembly, proteolytic competence in MALT1 is turned on and persists throughout the entire MALT1 activation process (indicated with scissors). MALT1 substrates identified to date can all be cleaved as part of CBM or TM complexes (data not shown). Only for MALT1B is proteolytic function severely impaired after self-cleavage at R781. Self-cleavage of MALT1A at R149 is favored by strong interaction with TRAF6 and represents a feed-forward mechanism that releases a highly functional MT complex from a transient CBMT complex and actively contributes to MALT1 turnover. This mechanism is less favored in MALT1B which cannot interact strongly with TRAF6. Self-cleavage of MALT1A at R781 weakens the interaction with TRAF6. In MALT1B, cleavage at the equivalent site (R770) abrogates interaction with TRAF6, “locking” the truncated MALT1 in the CBM complex.

Once MALT1 has become an active protease, subsequent regulation seems to affect mostly scaffolding properties with resulting consequences on MALT1 proteolytic function. A recent report showed that expression of MALT1 isoforms is differentially regulated in T lymphocytes. Following activation, upregulation of MALT1A expression is believed to enhance scaffolding with TRAF6 and allow for optimal T-cell activation [20]. Extending on these findings, our work has shown that C-terminal self-cleavage has an impact on scaffolding of MALT1, as seen in reduced TRAF6-dependent (i) production of N-terminally cleaved MALT1 (Fig 7B), (ii) cleavage of CYLD (Fig 7C) and (iii) down-regulation of MALT1 (Fig 7C and 7D). Functional consequences were profound for the B isoform, as mentioned above. By accentuating the scaffolding differences between the two MALT1 isoforms, auto-cleavage reactions may therefore provide an additional means of regulation for tight control of cellular activation cascades (Fig 10).

Control by TRAF6 of MALT1 auto-proteolysis appears to affect the fate of the CBM complex. On the one hand, by inducing N-terminal cleavage of MALT1, TRAF6 disrupts the BCL10-MALT1 interaction [18, 25, 47] (Fig 10). On the other hand, our data suggest that TRAF6 is able to accelerate the turnover of MALT1 in a MALT1-protease dependent manner (Fig 10). When TRAF6 was co-expressed with MALT1, MALT1A was profoundly down-regulated, significantly more than MALT1B whereas both C-terminally cleaved isoforms were remarkably stable (Fig 7B and 7C). This indicates that the C-terminus of MALT1 plays a major role in regulating MALT1 stability. Under conditions of limited proteolytic activity (MALT1-K644R) or absence of catalytic activity (MALT1-C464A), down regulation of MALT1 by TRAF6 was abolished (Fig 7D, S7A and S7B Fig). That a direct interaction between MALT1 and TRAF6 is required was shown by using the TRAF6-binding deficient MALT1 mutant MALT1-4E/A that resisted down-regulation in the presence of TRAF6 (Fig 7A). TRAF6 levels might also become downregulated upon interaction with MALT1 (see Figs 5–8) although more data are required to prove this. Down-regulation of endogenous MALT1 protein levels was also evident in the OCI-Ly3 cell line characterized by chronic CBM-driven NF-κB signaling. Levels of MALT1 could be significantly up-regulated in the presence of z-VRPR-fmk (Fig 3A). Altogether, these observations suggest that MALT1, as a protease, may play an active role, not only during the triggering phase of NF-κB cascades, but also for resolution, dampening CBM signaling (Fig 10).

In a previous in vivo study, mice featuring CD4-selective deletion of TRAF6 developed a multi-organ inflammatory disease which suggested that TRAF6 may in fact negatively regulate T-cell activation by controlling the degree of suppression by regulatory T cells [51]. In the context of the current work, one might speculate that absence of TRAF6, by preventing or limiting self-cleavage of MALT1 at R149, led to stabilization of the CBM complex after T cell activation, resulting in signaling capabilities not adequately controlled by regulatory cells. The hypothesis that TRAF6, by controlling MALT1 auto- proteolytic activity, might function in the maintenance of peripheral tolerance, would be worth further investigation.

Finally, the regulation of MALT1 by mono-ubiquitination remains an intriguing process. Our study confirmed that K644 is a major site for regulation of MALT1 function. Kinetic measurements in Jurkat T cells (Fig 3A) and human primary T cells (Fig 3B) also confirmed mono-ubiquitination to be an early event that is initially not dependent on MALT1 proteolytic activity, but starts to be down regulated as proteolytic potential builds up. Blockade of proteolytic function therefore leads to accumulation of mono-ubiquitinated MALT1 (Fig 3A and 3B) [19]. However, it seems that C-terminal auto-cleaved species of MALT1 may continue to accumulate when mono-ubiquitination of MALT1 is no longer detectable. As the MALT1 K644R retains a low level of proteolytic capability (Fig 8D) including C-terminal auto-proteolysis potential, at least in the context of the CBM (Fig 9B), mono-ubiquitination might not be strictly required for C-terminal auto-cleavage to occur. Clearly, the sequence of events that shape the function of MALT1 will need to be further addressed. In particular, ubiquitination/deubiquitination of MALT1 and more generally of the CBMT complex is still incompletely understood despite some recent advances [52, 53]. Based on our data with the MALT1- 4E/A mutant protein (Fig 5C) and the dominant negative TRAF6 522–689 protein (Fig 6B), it is unlikely that TRAF6 is responsible for mono-ubiquitination of MALT1. Therefore, the quest for the relevant ubiquitin ligase remains open.

## Supporting Information

S1 Fig Ectopic CARD11-BCL10-MALT1 (CBM) reconstitution in HEK293 cells triggers MALT1 post-translational modifications. A CBM reconstitution experiment was performed as described in 2A. This figure shows evidence for BCL10 cleavage ─ only when the full CBM is reconstituted ─ by using an affinity-purified rabbit polyclonal antibody that was raised against the C-terminal BCL10 neo-epitope (FLPLRSR) generated upon cleavage by MALT1. BCL10^Δ5^ refers to C-terminally-cleaved BCL10, which lacks 5 amino-acid residues.
(TIF)

S2 Fig Ectopic CBM reconstitution with CARD9 also triggers MALT1 post-translational modifications. CBM complex components ─ either CARD9 long or short isoforms, or CARD11-L244P ─ together with BCL10 and FLAG-MALT1 ─ were ectopically expressed in HEK293 cells (CBM reconstitution assay). Cells were treated or not with 100 μM z-VRPR-fmk. Twenty four hours after transfection, full lysates were harvested and analyzed with anti-CARD9 (Cell Signaling Technology #12416, Rabbit polyclonal), anti-CARD11 and anti- FLAG antibodies (see main text). The white arrow head points to auto-cleaved MALT1A at R781 (faster migrating species), the black one to mono-ubiquitinated MALT1A (slower running species), as described in the main text. The band indicated with (*) was detected with the anti -CARD11 antibody. The CARD9 expressing plasmids were obtained from GeneCopoeia, (pReceiver-M02 vector).
(TIF)

S3 Fig Ectopic CBM reconstitution triggers MALT1 ubiquitination at K644. **(A)** CBM reconstitution assay using MALT1 WT in the absence or presence of 100 μM z-VRPR-fmk. Cell lysates were subjected to SDS-PAGE and immunoblot analysis using anti-FLAG to detect MALT1 (top panel) and anti-ubiquitin (BML-PW8810-0100, middle panel) antibodies. The bottom panel shows an anti-tubulin immunoblot as loading control **(B)** CBM reconstitution assay using MALT1 WT in the absence or presence of 100 μM z-VRPR-fmk. Cell lysates were subjected to SDS-PAGE and immunoblot analysis using a mouse anti-Ubiquitin antibody (BML-PW8810-0100, red) and a rabbit anti-C-ter MALT1 antibody (Cell Signaling Technology #2494, green). **(C)** 10 μl lysates containing modified MALT1-C464A were incubated for 30 min at 30°C with PBS as control or with either 111 units alkaline Phosphatase (aP, Sigma #P0114) or 0.63 μg ubiquitin specific protease 2 (USP2, catalytic domain, Enzo lifesciences, #BML-UW9850). The reaction was stopped by addition of 5 μl sample buffer. Samples were resolved by SDS-PAGE and analyzed by immunoblotting using the antibodies described above. The black arrow head points to mono-ubiquitinated MALT1 (slower running species), as described in the main text.
(TIF)

S4 Fig MALT1 protease inhibition stabilizes MALT1 and BCL10. CBM reconstitution assays were set up in the absence or presence of 100 μM z-VRPR-fmk. Cycloheximide 200 μM was subsequently added to block protein synthesis 10h or 6h before harvest, or at time of harvest (control). Immunoblotting with anti-FLAG antibody (MALT1), anti-BCL10 (ep605y) and anti-Tubulin (loading control) is shown.
(TIF)

S5 Fig Auto-cleavage and ubiquitination of MALT1 in mouse lymphocytes. Purified WT or protease-deficient -MALT1 knock-in T cells (mouse) (37) were pre-treated for 30 min with 5 μM MG-132 and stimulated or not (control) for 2h30 min with 10 ng/ml PMA and 1 μM ionomycin. Post-nuclear lysates were resolved by SDS-PAGE and analyzed by immunoblotting using an anti-MALT1 antisera. The MALT1 faster and slower migrating species, described in the main text, are indicated with a white and a black arrow head, respectively.
(TIF)

S6 Fig Impact of TRAF family proteins on MALT1A auto-cleavage at R149. **(A)** A TM reconstitution assay was performed using FLAG-TRAF2, 3xFLAG-TRAF3 or FLAG-TRAF6. Immunoblot analysis with anti-FLAG antibody is shown. TRAF6 (but neither TRAF2 nor TRAF3) induces auto-cleavage (white arrow head) and mono-ubiquitination in the presence of z-VRPR-fmk (black arrow head). **(B)** A TM reconstitution assay was performed using FLAG-TRAF6 WT, the FLAG-TRAF6-F118A or the FLAG-TRAF6-K124R mutant constructs. Western Blot analysis with anti-FLAG antibody is shown.
(TIF)

S7 Fig The MALT1 4E/A mutant protein is proteolysis competent in a CBM assay, not in a TM assay. CBM (A) and TM (B) reconstitution assays in HEK293 cells were performed in the presence of co-expressed CYLD with MALT1 WT and mutant forms of isoform A, as labelled. Anti-FLAG Western Blot analyses show MALT1 C-terminal auto-cleavage bands (white arrow heads) as well as CYLD full length (*fl*) and cleaved fragment (*cl*) levels. An example of anti-CYLD (green) and anti-MALT1 immunoblots (red) from an alternative experiment is provided in (C).
(TIF)

S8 Fig A20, CYLD, HOIP and MALT1 proteolytic activity are not responsible for MALT1 mono-ubiquitination/de-mono-ubiquitination. TM reconstitution assays in HEK293 cells were performed with MALT1A-C464A (A) or MALT1A-WT (B), in the presence of co-expressed A20, CYLD, HOIP-WT or catalysis-deficient HOIP-C885A (A) or a FLAG-tagged ubiquitin-expressing plasmid encoding WT-ubiquitin or ubiquitin -C72A,C74A (B). Immunoblots with anti-A20, anti-HOIP (Abcam, ab46322, rabbit polyclonal) and anti-FLAG antibodies (A) or anti-MALT1 (Cell Signaling Technology, #2494, rabbit polyclonal) and anti-FLAG antibodies (B). The black arrow heads refer to mono-ubiquitinated MALT1. Two or three black arrow heads in a row are used to indicate additional ubiquitinated species of MALT1.
(TIF)

S1 Table Oligonucleotides for mutagenesis reactions. (DOCX)

S2 Table Conservation of MALT1 self-cleavage sites across species. (DOCX)
