# Supplementary figures and images for: Two Antagonistic MALT1 Auto-Cleavage Mechanisms Reveal a Role for TRAF6 to Unleash MALT1 Activation
Source: PLoS One. 2017 Jan 4;12(1):e0169026. doi: 10.1371/journal.pone.0169026 (PMC5214165; doi:10.1371/journal.pone.0169026)

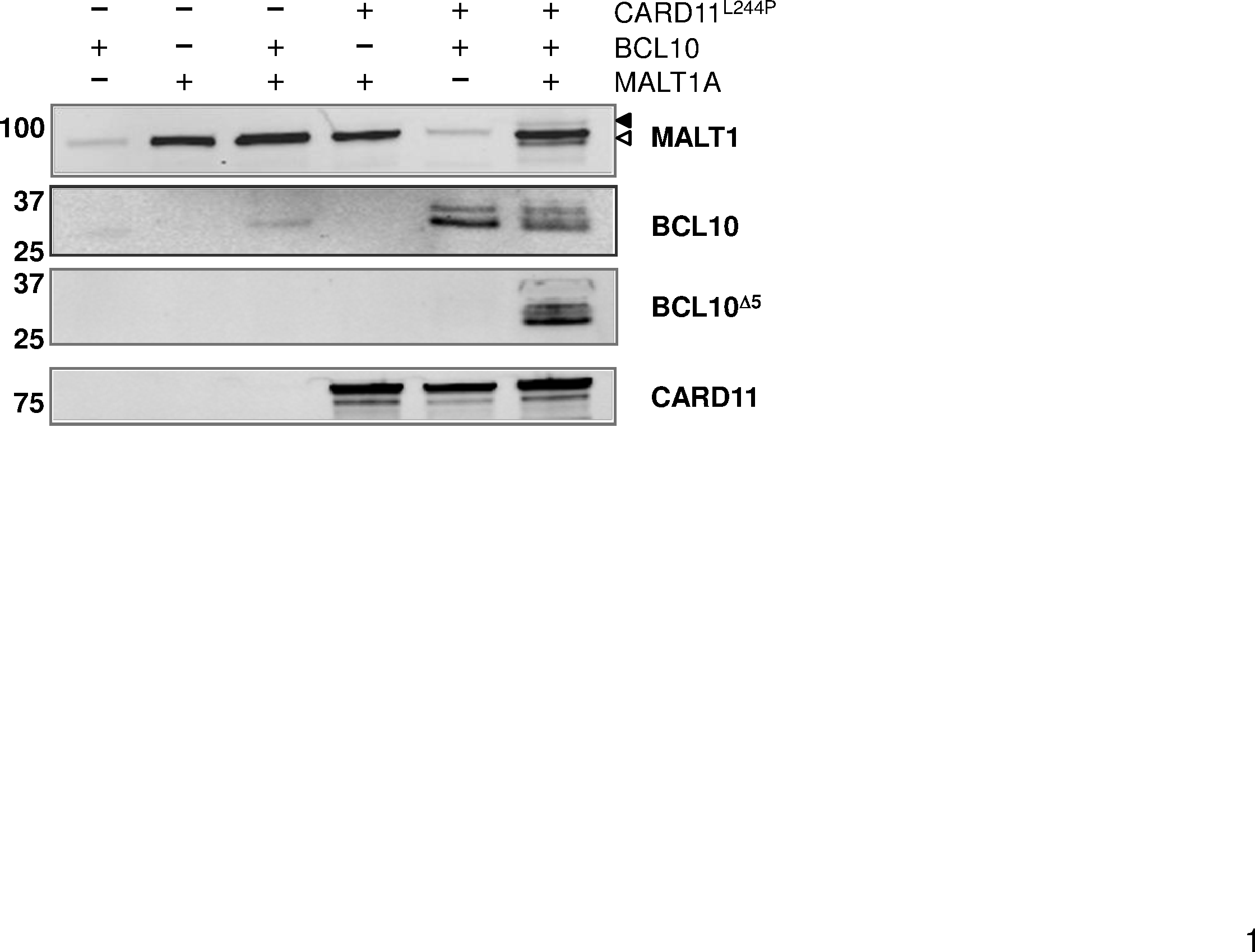

Supplement: S1 Fig — A CBM reconstitution experiment was performed as described in 2A. This figure shows evidence for BCL10 cleavage ─ only when the full CBM is reconstituted ─ by using an affinity-purified rabbit polyclonal antibody that was raised against the C-terminal BCL10 neo-epitope (FLPLRSR) generated upon cleavage by MALT1. BCL10Δ5 refers to C-terminally-cleaved BCL10, which lacks 5 amino-acid residues. (TIF) [file pone.0169026.s001.tif]

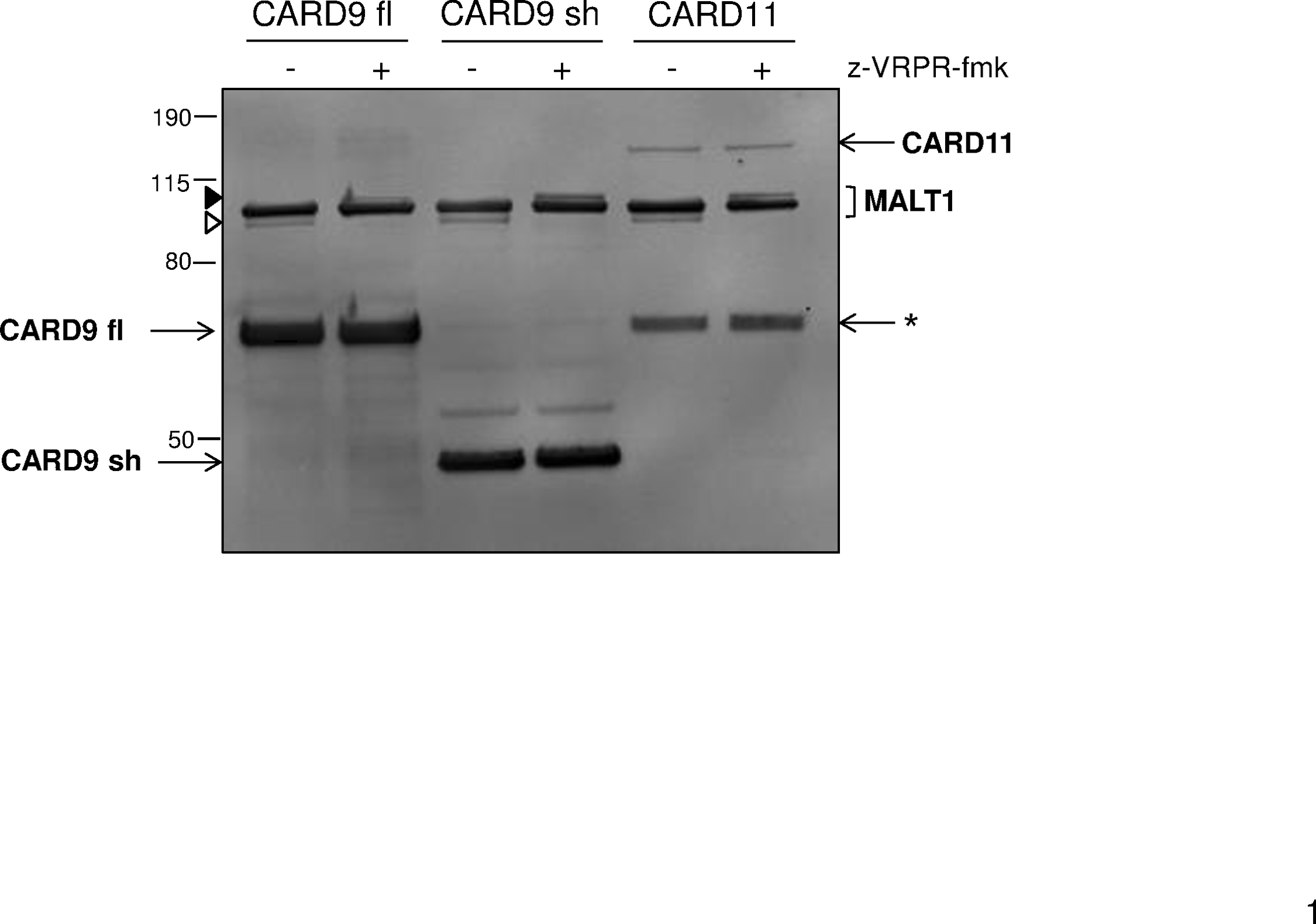

Supplement: S2 Fig — CBM complex components ─ either CARD9 long or short isoforms, or CARD11-L244P ─ together with BCL10 and FLAG-MALT1 ─ were ectopically expressed in HEK293 cells (CBM reconstitution assay). Cells were treated or not with 100 μM z-VRPR-fmk. Twenty four hours after transfection, full lysates were harvested and analyzed with anti-CARD9 (Cell Signaling Technology #12416, Rabbit polyclonal), anti-CARD11 and anti- FLAG antibodies (see main text). The white arrow head points to auto-cleaved MALT1A at R781 (faster migrating species), the black one to mono-ubiquitinated MALT1A (slower running species), as described in the main text. The band indicated with (*) was detected with the anti -CARD11 antibody. The CARD9 expressing plasmids were obtained from GeneCopoeia, (pReceiver-M02 vector). (TIF) [file pone.0169026.s002.tif]

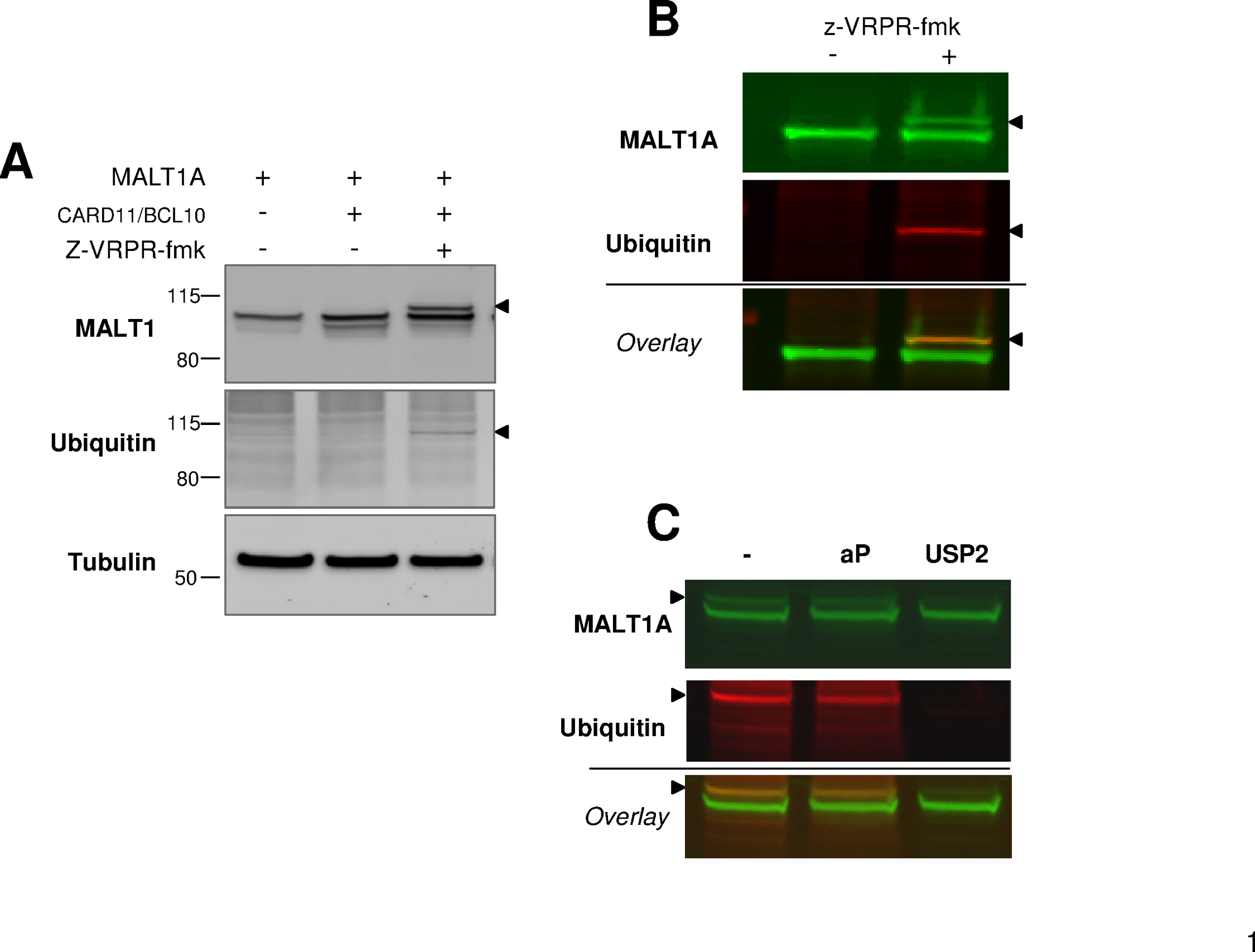

Supplement: S3 Fig — (A) CBM reconstitution assay using MALT1 WT in the absence or presence of 100 μM z-VRPR-fmk. Cell lysates were subjected to SDS-PAGE and immunoblot analysis using anti-FLAG to detect MALT1 (top panel) and anti-ubiquitin (BML-PW8810-0100, middle panel) antibodies. The bottom panel shows an anti-tubulin immunoblot as loading control (B) CBM reconstitution assay using MALT1 WT in the absence or presence of 100 μM z-VRPR-fmk. Cell lysates were subjected to SDS-PAGE and immunoblot analysis using a mouse anti-Ubiquitin antibody (BML-PW8810-0100, red) and a rabbit anti-C-ter MALT1 antibody (Cell Signaling Technology #2494, green). (C) 10 μl lysates containing modified MALT1-C464A were incubated for 30 min at 30°C with PBS as control or with either 111 units alkaline Phosphatase (aP, Sigma #P0114) or 0.63 μg ubiquitin specific protease 2 (USP2, catalytic domain, Enzo lifesciences, #BML-UW9850). The reaction was stopped by addition of 5 μl sample buffer. Samples were resolved by SDS-PAGE and analyzed by immunoblotting using the antibodies described above. The black arrow head points to mono-ubiquitinated MALT1 (slower running species), as described in the main text. (TIF) [file pone.0169026.s003.tif]

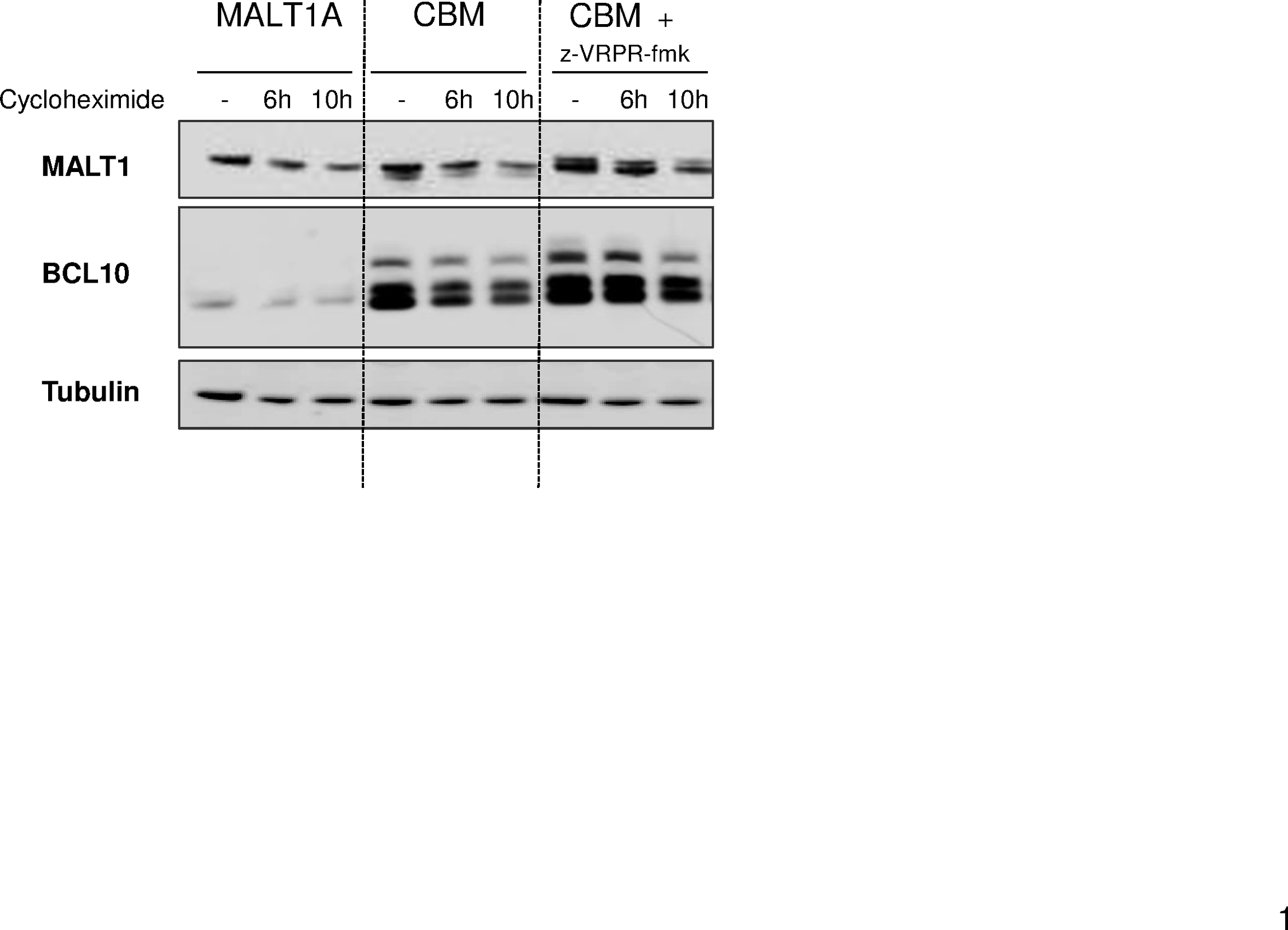

Supplement: S4 Fig — CBM reconstitution assays were set up in the absence or presence of 100 μM z-VRPR-fmk. Cycloheximide 200 μM was subsequently added to block protein synthesis 10h or 6h before harvest, or at time of harvest (control). Immunoblotting with anti-FLAG antibody (MALT1), anti-BCL10 (ep605y) and anti-Tubulin (loading control) is shown. (TIF) [file pone.0169026.s004.tif]

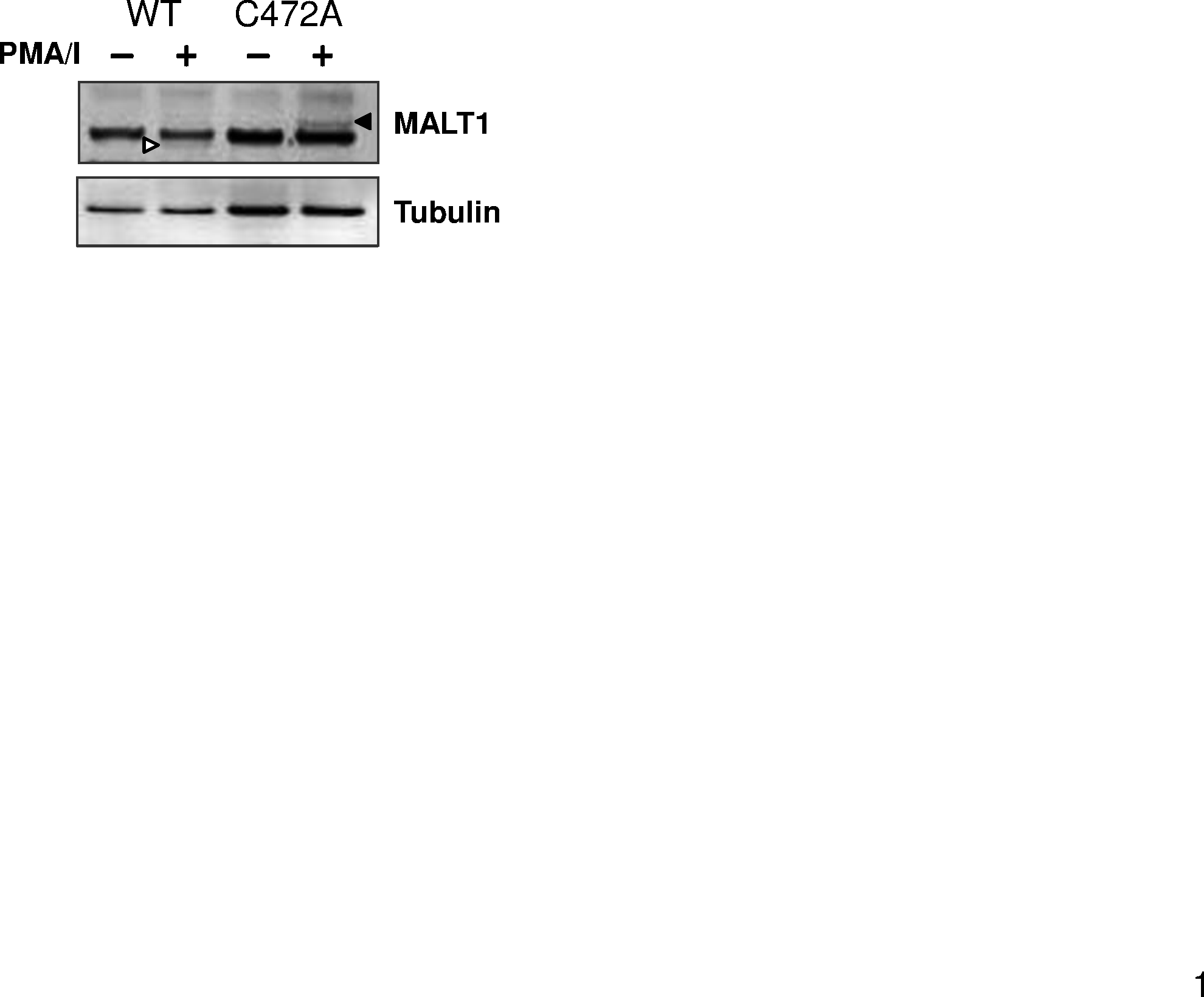

Supplement: S5 Fig — Purified WT or protease-deficient -MALT1 knock-in T cells (mouse) (37) were pre-treated for 30 min with 5 μM MG-132 and stimulated or not (control) for 2h30 min with 10 ng/ml PMA and 1 μM ionomycin. Post-nuclear lysates were resolved by SDS-PAGE and analyzed by immunoblotting using an anti-MALT1 antisera. The MALT1 faster and slower migrating species, described in the main text, are indicated with a white and a black arrow head, respectively. (TIF) [file pone.0169026.s005.tif]

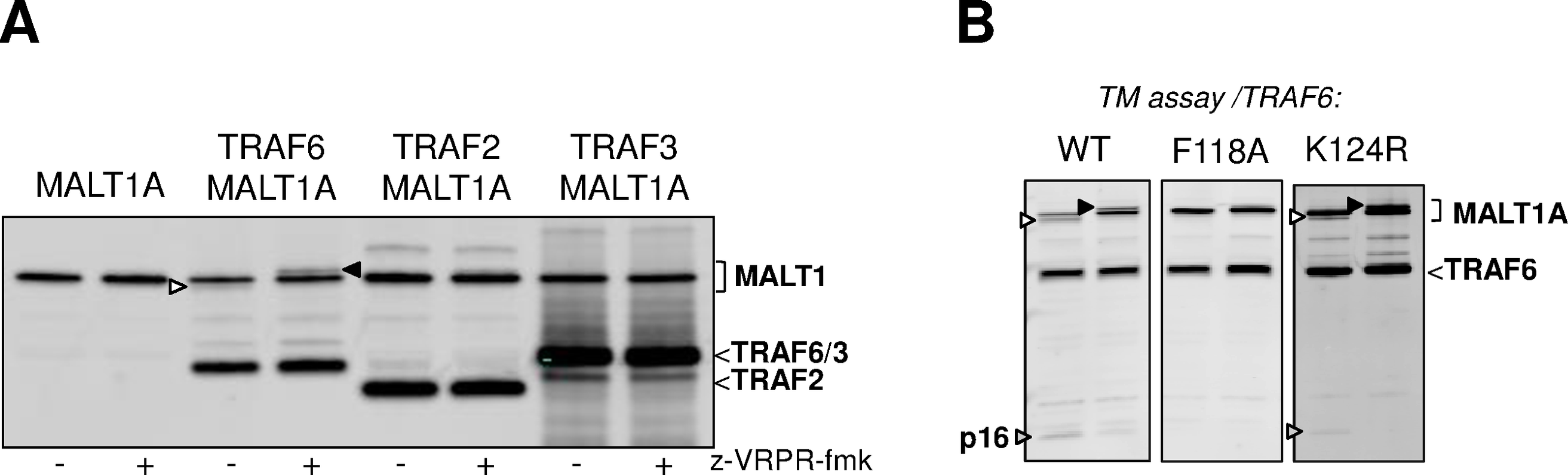

Supplement: S6 Fig — (A) A TM reconstitution assay was performed using FLAG-TRAF2, 3xFLAG-TRAF3 or FLAG-TRAF6. Immunoblot analysis with anti-FLAG antibody is shown. TRAF6 (but neither TRAF2 nor TRAF3) induces auto-cleavage (white arrow head) and mono-ubiquitination in the presence of z-VRPR-fmk (black arrow head). (B) A TM reconstitution assay was performed using FLAG-TRAF6 WT, the FLAG-TRAF6-F118A or the FLAG-TRAF6-K124R mutant constructs. Western Blot analysis with anti-FLAG antibody is shown. (TIF) [file pone.0169026.s006.tif]

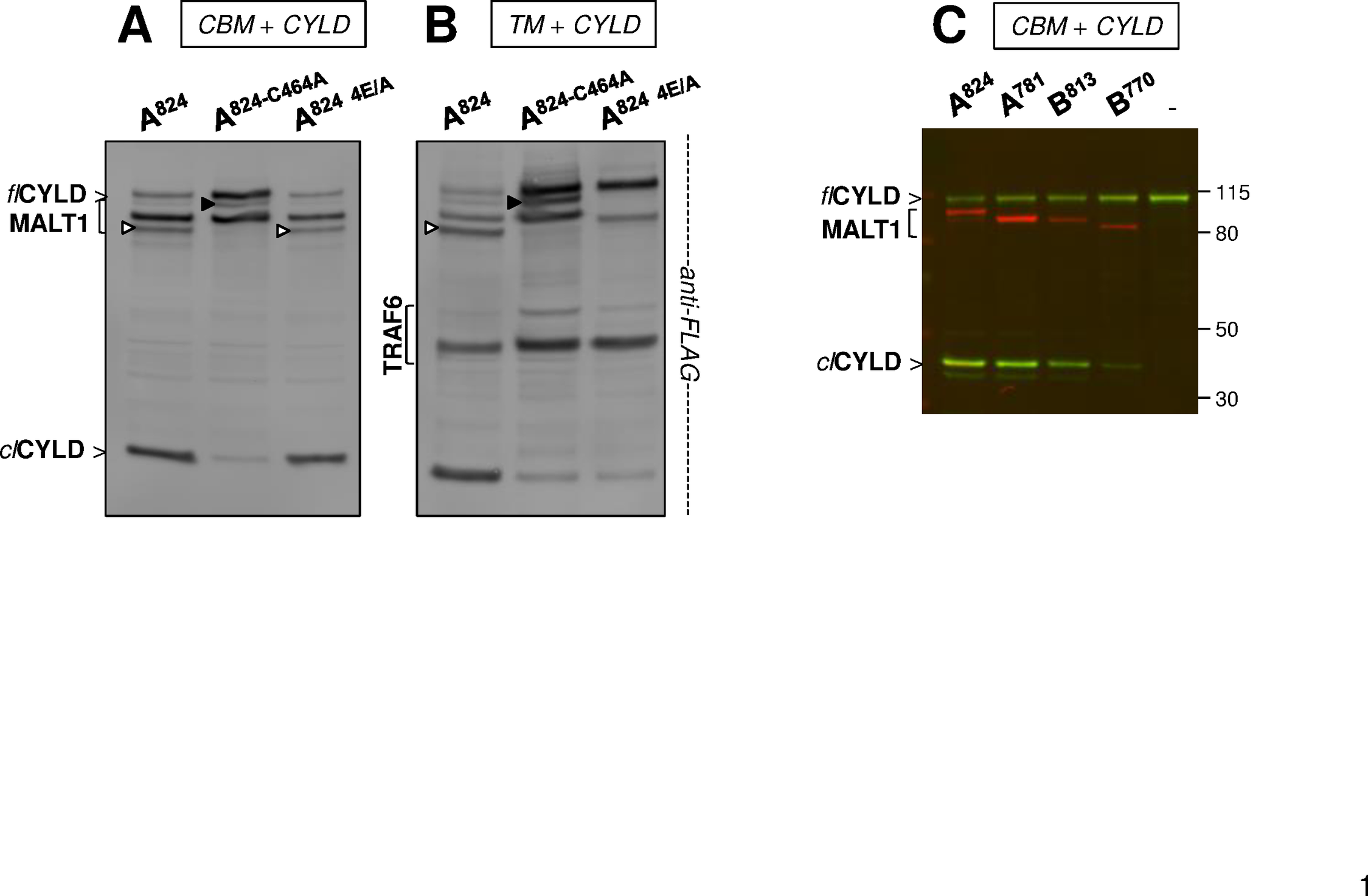

Supplement: S7 Fig — CBM (A) and TM (B) reconstitution assays in HEK293 cells were performed in the presence of co-expressed CYLD with MALT1 WT and mutant forms of isoform A, as labelled. Anti-FLAG Western Blot analyses show MALT1 C-terminal auto-cleavage bands (white arrow heads) as well as CYLD full length (fl) and cleaved fragment (cl) levels. An example of anti-CYLD (green) and anti-MALT1 immunoblots (red) from an alternative experiment is provided in (C). (TIF) [file pone.0169026.s007.tif]

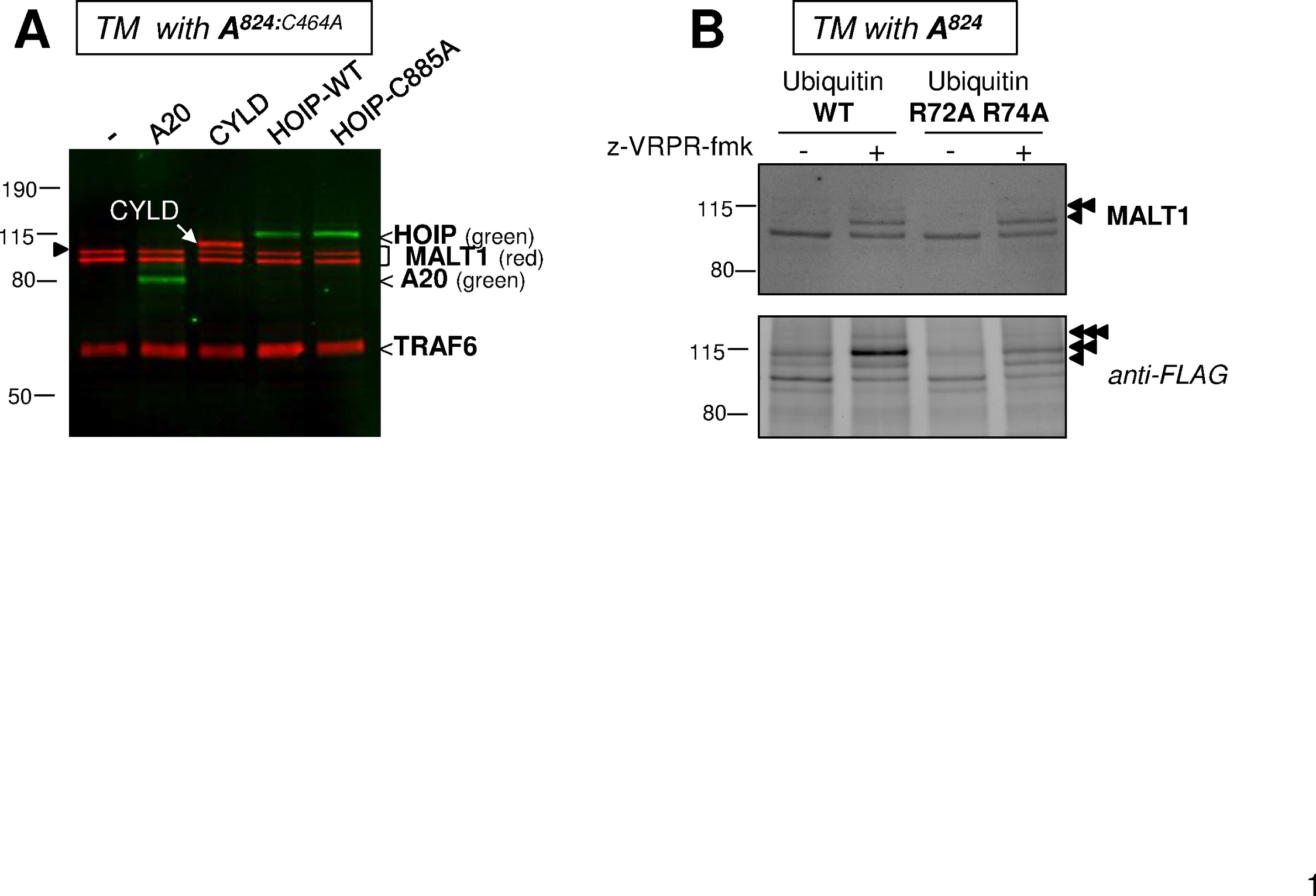

Supplement: S8 Fig — TM reconstitution assays in HEK293 cells were performed with MALT1A-C464A (A) or MALT1A-WT (B), in the presence of co-expressed A20, CYLD, HOIP-WT or catalysis-deficient HOIP-C885A (A) or a FLAG-tagged ubiquitin-expressing plasmid encoding WT-ubiquitin or ubiquitin -C72A,C74A (B). Immunoblots with anti-A20, anti-HOIP (Abcam, ab46322, rabbit polyclonal) and anti-FLAG antibodies (A) or anti-MALT1 (Cell Signaling Technology, #2494, rabbit polyclonal) and anti-FLAG antibodies (B). The black arrow heads refer to mono-ubiquitinated MALT1. Two or three black arrow heads in a row are used to indicate additional ubiquitinated species of MALT1. (TIF) [file pone.0169026.s008.tif]
