# Supplementary material for: Two Antagonistic MALT1 Auto-Cleavage Mechanisms Reveal a Role for TRAF6 to Unleash MALT1 Activation
Source: PLoS One. 2017 Jan 4;12(1):e0169026. doi: 10.1371/journal.pone.0169026 (PMC5214165; doi:10.1371/journal.pone.0169026)
Supplement: S1 Table — (DOCX) [file pone.0169026.s009.docx]

| **Proteins** | **Mutations** | **Sense** | **Primers** |
| --- | --- | --- | --- |
| **MALT1A** | **R149A** | **F** | GTCACGGCTCTTCTGCCGCAACTGGACATCCTTT |
|  |  | **R** | CAGCAGCTCTTCAGGCGCAACACAGTTTCACAAAC |
|  | **(150-824)** | **F** | GCCGGCTCTTCTGCAACTGGACATCCTTTTGTTC |
|  |  | **R** | CGCAGCTCTTCATGCTCCTGGACCCTGGAACAGAAC |
|  | **C464A** | **F** | GACTTAATGTGTTCTTATTGGATATGGCTAGGAAAAGAAATGACTACGATGATA |
|  |  | **R** | TATCATCGTAGTCATTTCTTTTCCTAGCCATATCCAATAAGAACACATTAAGTC |
|  | **K644R** | **F** | GATCTAGATATTGATCCAAGAGATGCAAATAAAGGCACACC |
|  |  | **R** | GGTGTGCCTTTATTTGCATCTCTTGGATCAATATCTAGATC |
|  | **R781A** | **F** | AGATAGCTGTCATTGCAGCGCGACTCCAGATGCATTTATT |
|  |  | **R** | AATAAATGCATCTGGAGTCGCGCTGCAATGACAGCTATCT |
|  | **A(1-781) / B(1-770)** | **F** | GATAGCTGTCATTGCAGCCGGTAACCAGATGCATTTATTTCAAG |
|  |  | **R** | CTTGAAATAAATGCATCTGGTTACCGGCTGCAATGACAGCTATC |
|  | **R800A** | **F** | CCATGCTTCATGTCATTTTAGTGCAAGTAATGTGCCAGTAGAGACA |
|  |  | **R** | TGTCTCTACTGGCACATTACTTGCACTAAAATGACATGAAGCATGG |
|  | **C70A** | **F** | CTGAAAGTGACTGCTGTGTG |
|  |  | **R** | CCTTGGACAATCCTTCAGAATG |
| **TRAF6** | **F118A** | **F** | GTTGACAATGAAATACTGCTGGAAAATCAACTAGCTCCAGACAATTTTGCAAA |
|  |  | **R** | TTTGCAAAATTGTCTGGAGCTAGTTGATTTTCCAGCAGTATTTCATTGTCAAC |
|  | **K124R** | **F** | AATCAACTATTTCCAGACAATTTTGCAAGACGTGAGATTCTTTCTCT |
|  |  | **R** | AGAGAAAGAATCTCACGTCTTGCAAAATTGTCTGGAAATAGTTGATT |
