# Supplementary material for: Two Antagonistic MALT1 Auto-Cleavage Mechanisms Reveal a Role for TRAF6 to Unleash MALT1 Activation
Source: PLoS One. 2017 Jan 4;12(1):e0169026. doi: 10.1371/journal.pone.0169026 (PMC5214165; doi:10.1371/journal.pone.0169026)
Supplement: S2 Table — (DOCX) [file pone.0169026.s010.docx]

Homo sapiens (human)

Pan Troglodyte (chimpanzee)

Pongo abelii (orang-utan)

Otolemur garnettii (galago)

Callithrix jacchus (marmoset)

Macaca mulatta (rhesus macaque)

Papio anubis(olive baboon)

Gorilla Gorilla

Mus Musculus (mouse)

Sus Scrofa (wild boar)

Cavia porcellus (guinea pig)

Canis familiaris (dog)

Felis catus (cat)

Ovis aries (sheep)

Equus caballus (horse)

Loxodonta africana (elephant)

Bos taurus(bovine)

Sarcophilus harrisii (Tasmanian devil)

Mustela putorius furo (ferret)

Myotis lucifugus (brown bat)

Monodelphis domestica (opossum)

Pelodiscus sinensis (turtle)

Meleagris gallopavo (common turkey)

Ficedula albicollis (flycatcher)

Taeniopygia guttata (Zebra finch)

Anas platyrhynchos (mallard)

**R781**

CHCS**R**TP---DAFISSF

CHCS**R**TP---DAFISSF

CHCS**R**TP---DAFISSF

CHCS**R**TP---DAFISSY

CHCS**R**TP---DAFISSF

CHCS**R**TP---DAFISSF

CHCS**R**TP---DAFISSF

CHCS**R**TP---DAFISSF

CHCS**R**TP---HTFISNY

CQCS**R**TA---DAFISSH

CHCS**R**TPNASNAFISSY

CRCS**R**TA---DAFMSSH

CHCS**R**TA---DAFISSP

CRCS**R**TA---DTFVSSH

CRCS**R**TA---DAFLSSH

CHCS**R**TP---DPFVSSY

CRCS**R**TA---DAFVSSH

CHCNQTP---DTLITSH

CRCS**R**MA---DAFISSH

CQCS**R**TA---DAFISSH

CHCNQSS---DTLISSH

CHCIMAL---N-ITSRR

CSCNGTS---RMLASRH

CSCNGTS---STLASRR

-----------------

CSCNGTS---RILASRH

LCC**R**ATGHPF

LCC**R**ATGHPF

LCC**R**ATGHPF

LCC**R**ATGHPF

LCC**R**ATGHPF

LCC**R**ATGHPF

LCC**R**ATGHPF

LCC**R**ATGHPF

LCC**R**ATGHPF

LCC**R**ATGHPF

LCC**R**ATGHPF

LCC**R**ATGHPF

LCC**R**ATGHPF

LCC**R**ATGHPF

LCC**R**ATGHPF

LCC**R**ATGHPF

LCC**R**ATGHPF

LCC**R**ATGHPF

LCC**R**ATGHPF

LCC**R**ATGHPF

FSICQLCXPF

LCCWATGLPF

LCCWATGHPF

LCCWAAGHPF

LCCWATGLPF

LCCWATGHPF

**R149**
